# Inhibition of insulin-like growth factors increases production of CXCL9/10 by macrophages and fibroblasts and facilitates CD8^+^ cytotoxic T cell recruitment to pancreatic tumours

**DOI:** 10.3389/fimmu.2024.1382538

**Published:** 2024-08-05

**Authors:** Patrick Freeman, Gaia Bellomo, Lucy Ireland, Maidinaimu Abudula, Teifion Luckett, Michael Oberst, Ruth Stafferton, Paula Ghaneh, Chris Halloran, Michael C. Schmid, Ainhoa Mielgo

**Affiliations:** ^1^ Department of Molecular and Clinical Cancer Medicine, University of Liverpool, Liverpool, United Kingdom; ^2^ Department of Oncology Research, AstraZeneca, One Medimmune Way, Gaithersburg, MD, United States

**Keywords:** pancreatic cancer, tumour microenvironment, CD8^+^ T cell, IGF, macrophage, fibroblast, CXCL9/10

## Abstract

Pancreatic ductal adenocarcinoma (PDAC) is a highly lethal malignancy with an urgent unmet clinical need for new therapies. Using a combination of *in vitro* assays and *in vivo* preclinical models we demonstrate that therapeutic inhibition of the IGF signalling axis promotes the accumulation of CD8^+^ cytotoxic T cells within the tumour microenvironment of PDAC tumours. Mechanistically, we show that IGF blockade promotes macrophage and fibroblast production of the chemokines CXCL9 and CXCL10 to facilitate CD8^+^ T cell recruitment and trafficking towards the PDAC tumour. Exploring this pathway further, we show that IGF inhibition leads to increased STAT1 transcriptional activity, correlating with a downregulation of the AKT/STAT3 signalling axis, in turn promoting *Cxcl9* and *Cxcl10* gene transcription. Using patient derived tumour explants, we also demonstrate that our findings translate into the human setting. PDAC tumours are frequently described as “immunologically cold”, therefore bolstering CD8^+^ T cell recruitment to PDAC tumours through IGF inhibition may serve to improve the efficacy of immune checkpoint inhibitors which rely on the presence of CD8^+^ T cells in tumours.

## 1 Introduction

Pancreatic ductal adenocarcinoma (PDAC), accounting for > 85% of all pancreatic cancers (1), is a devastating disease with current treatment outcomes remaining notoriously poor and current treatment modalities lagging far behind those of other solid malignancies. PDAC is currently the 5^th^ leading cause of cancer-related death in the UK and is projected to become the second leading cause of cancer death within the next decade, having already surpassed breast cancer in the United States (2). Late diagnosis, early (and high) rates of metastasis and therapeutic resistance (3, 4) are all key driving factors for disease lethality, with only 11% of patients achieving a 5-year survival (5). As such, PDAC poses a major public health concern, and greater emphasis needs to be made on delineating the complex pathophysiology underpinning this deadly disease and developing new therapeutic interventions.

A defining hallmark of PDAC, and one of the major contributing factors for therapeutic resistance, is the complex nature of its tumour microenvironment (TME) which has been explored by us and others (3, 6–11). The TME is a complex environment in which various non-cancerous cell populations co-exist, co-evolve and interact with tumour cells, having a profound impact on cancer progression (12). PDAC has been described as “immunologically cold”, with tumours generally displaying very limited numbers of cytotoxic CD8^+^ T cells (13). Compounding this, the pancreatic TME is highly infiltrated by a variety of immunosuppressive cells, including: “M2-like” tumour-associated macrophages (TAMs); cancer-associated fibroblasts (CAFs) and regulatory T cells (Tregs) (14–17). Collectively, these cells work in concert to mitigate the function of cytotoxic CD8^+^ T cells through the secretion of a host of immunosuppressive factors, resulting in a poor anti-tumour immune response (12, 18). Additionally, immune/stromal derived factors and the characteristically dense desmoplastic reaction of PDAC serve to impede CD8^+^ accumulation within the pancreatic TME (6, 19–23). With CD8^+^ T cells being the backbone of emerging immunotherapeutic strategies such as immune checkpoint inhibitors (ICIs) and cancer treatment vaccines, their low abundance within the pancreatic TME can explain the overall lack of success of these therapeutic modalities in the treatment of PDAC (24–28). Indeed, retrospective analysis of surgically resected specimens displays that a greater abundance of CD8^+^ T cells alongside high levels of tumour-specific antigens within the TME confers a survival advantage in PDAC patients (29). Any therapy that is able to bolster the accumulation of CD8^+^ T cells within the TME of pancreatic tumours therefore has the potential to improve anti-tumour immunity.

Previous work published by our group and others shows an important role for the IGF signalling pathway in promoting cancer progression and resistance to therapies in a multitude of human malignancies, including PDAC, triple-negative breast cancer (TNBC), colorectal, bladder and ovarian cancer (3, 30–33). Importantly, we previously found that IGF signalling through stromal components including tumour associated macrophages (TAMs) and cancer associated fibroblasts (CAFs) contributes to chemoresistance in the context of both PDAC and TNBC, with blockade of the IGF signalling pathway sensitising these tumours to gemcitabine and paclitaxel respectively (3, 30). Since IGF signalling is known to influence tumour progression through extrinsic mechanisms via the tumour microenvironment (TME) (34–37), we sought to further characterise changes in the PDAC TME upon blockade of the IGF signalling axis. In addition to this, evidence suggests an emerging immunomodulatory role of the IGF signalling axis, which has been subject of a recent review by Pellegrino et al. (38).

In the present article we show that therapeutic inhibition of the IGF signalling axis leads to an increased accumulation of CD8^+^ cytotoxic T cells within orthotopic PDAC tumours. Mechanistically, using a combination of *in vitro* assays, an *in vivo* preclinical model of PDAC and patient-derived tumour explants, we provide evidence that inhibition of IGF signalling promotes TAM/CAF production of the T cell chemokines CXCL9 and CXCL10 leading to an increase in CD8^+^ T cell recruitment and trafficking towards the pancreatic TME.

## 2 Materials and methods

### 2.1 Generation of primary KPC-derived pancreatic cancer cells

The murine pancreatic cancer cells KPC FC1242 were generated in the Tuveson lab (Cold Spring Harbor Laboratory, New York, USA), isolated from PDAC tumour tissues obtained from LSL-KrasG12D; LSL-Trp53R172H; Pdx1-Cre mice of a pure C57BL/6 background as described previously (39). FC1242^luc/zsGreen^ cells were generated using pHIV Luc-zsGreen lentiviral infection. FC1242^luc/zsGreen^ were cultured in DMEM supplemented with 10% FBS, 1% penicillin/streptomycin, at 37°C, 5% CO2 incubator.

### 2.2 Cell lines

Jurkat human T lymphocyte cells were cultured in in RPMI 1640 Medium supplemented with 10% FBS and 1% penicillin/streptomycin, at 37°C, 5% CO2 incubator. Jurkat cells were switched to DMEM, high glucose, GlutaMAX medium (Gibco, 10569010) with 10% FBS and 1% penicillin/streptomycin, at 37°C, 5% CO2 incubator for 24 hours prior to use in chemotaxis assays. All cells were routinely tested negative for the presence of Mycoplasma contamination.

### 2.3 Mice

6-8 weeks old female C57BL/6 mice were purchased from Charles River. All animal experiments were performed in accordance with current UK legislation under an approved project license PPL P16F36770 (M.C. Schmid). Mice were housed under specific pathogen-free conditions at the Biomedical Science Unit at the University of Liverpool.

### 2.4 Syngeneic orthotopic pancreatic cancer models

1 x 10^6^ primary KPC^luc/zsGreen^ (zsGreen) cells (FC1242^luc/zsGreen^) isolated from a pure C57Bl/6 background were implanted in 30 µL of Matrigel (VWR, 734-0269) into the pancreas of immunocompetent syngeneic C57Bl/6 six-to 8-week-old female mice, and tumours were established for two weeks before beginning treatment. Mice were administered intraperitoneally either IgG2 control antibody (BioXcell, BE0301) (60 mg/kg), or IGF-blocking antibody (MEDI-573) (60 mg/kg), kindly provided by Medimmune (AstraZeneca) at days 14, 17 and 21 post-implantation before harvesting and formalin fixation of both tumours and mesenteric lymph nodes, or collagenase digestion of tumours and subsequent CyTOF analysis at day 22. In a subsequent model, tumours were established as described above and mice administered a single intraperitoneal dose of IgG2 control antibody or IGF blocking antibody at day 23, before tumour harvesting and collagenase digestion and subsequent FACS analysis at day 25 post-implantation.

### 2.5 Analysis and quantification of immune cells in orthotopic PDAC tumours by mass cytometry

Single-cell suspensions from murine pancreatic tumours were prepared by mechanical and enzymatic disruption in Hank’s Balanced Salt Solution (Gibco, 24020091) with 1 mg/mL Collagenase P (Roche, 11213865001) as described (6, 9). Cells were centrifuged for 5 min at 1500 rpm, resuspended in HBSS and filtered through a 500 µm polypropylene mesh (Spectrum Laboratories). Cell suspensions were resuspended in 1mL 0.05% trypsin and incubated at 37°C, for 5 minutes. Suspensions were further enriched for immune cells by density gradient centrifugation using Histopaque-1083 (Sigma Aldrich) at 400x g for 30 minutes at room temperature without brakes. The cloudy band/interface containing the cells plus the bottom layer was transferred into new tube and gently washed with PBS. After one wash with PBS, the cell suspension was washed in double-deionised water (ddH2O; ≥ 18Ω)/Maxpar cell staining buffer (1:2 dilution). The pellet was resuspended in 1 ml of Maxpar cell staining buffer and cells were stained with Cell-ID ^195-^Cisplatin (Fluidigm) viability marker in Maxpar PBS (Fluidigm) for 5 min. Cells were washed with Maxpar cell staining buffer, blocked with Fc Block (BD Pharmingen, Clone 2.4G2) on ice for 10 min and metal-conjugated antibody cocktail added and incubated for 40 min at 4°C. See **Supplementary Table 1** for list of metal-conjugated antibodies. Antibodies were used at the concentrations recommended by the manufacturer. Cells were then washed twice in cell staining buffer and stained with 125 μM Intercalator ^191^Ir (Fluidigm) diluted 1:2,000 in Maxpar fix and perm buffer (Fluidigm) overnight at 4°C. Cells were then washed twice in Maxpar cell staining buffer followed by two washes in 18Ω distilled water (Fluidigm) and resuspended in 0.1X EQTM Four Element Calibration Beads (Fluidigm) prior acquisition on the Helios CyTOF system (Fluidigm). Samples were acquired at a rate of <500 events/sec. All generated FCS files were normalized and EQ beads standard (40). Data analysis was performed using Cytobank software (mrc.cytobank.org, v6.3 and v7.0, Beckman Coulter); manual gating was used to remove debris, identify single cells (^191^Ir+) and to distinguish between dead cells (^195^Pt+). Spanning-tree progression analysis for density-normalised events (SPADE) was performed on the data for mapping high dimensional relationships. Viable CD45^+^ singlets selected by manual gating were used for SPADE unsupervised clustering using equal sampling. Manual gating was then performed on the SPADE map created to determine cell population percentages.

### 2.6 Fluorescence activated cell sorting

Single-cell suspensions from murine pancreatic tumours were prepared by mechanical and enzymatic disruption as described above. Cells were filtered through a 70 μm cell strainer (Miltenyi) and resuspended in 0.5% BSA/PBS. Cells were blocked for 10 minutes on ice with purified rat anti-mouse CD16/CD32 (Mouse BD Fc Block™, BD biosciences, 553142) and then stained with Sytox^®^ blue viability marker (Invitrogen, S34857) and conjugated antibodies against anti-CD45-PE-Cy7 (1:100, Biolegend, clone 30-F11, 103114) and anti-F480-APC (1:100, Biolegend, clone BM8, 123116). Cells were incubated with antibodies for 45 min in the dark on ice and fluorescence activated cell sorting (FACS) was carried out using FACS Aria IIIu (BD Biosciences). Cells were sorted directly in RLT buffer + β-mercaptoethanol according to the manufacturer’s instruction for RNA isolation (Qiagen).

### 2.7 Generation of primary bone marrow derived macrophages, primary pancreatic fibroblasts, macrophage and fibroblasts conditioned media

Primary murine bone marrow derived macrophages (BMDMs) were generated by flushing the bone marrow from the femur and tibia of C57BL/6 mice followed by incubation for 5 days in DMEM containing 10% FBS and 10 ng/mL murine M-CSF (PeproTech, AF-315-02) as described (3). Primary pancreatic stellate cells were isolated from C57BL/6 mice pancreas by density gradient centrifugation, and were activated into fibroblasts by culturing them on uncoated plastic dishes in Iscove’s Modified Dulbecco’s Medium (IMDM) containing 10% FBS and 1% L-glutamine (Sigma Aldrich, G7513). Conditioned media was generated from both BMDMs and fibroblasts by culturing cells in serum free DMEM and IMDM respectively for 24 to 48 hours. For chemotaxis assays fibroblast conditioned media was generated from fibroblasts cultured in serum free RPMI 1640 medium. Supernatants were harvested and filtered using 0.22 µm filter and stored at 4°C until use.

### 2.8 siRNA knockdown of primary pancreatic fibroblasts

1 x 10^5^ primary pancreatic fibroblasts were treated in 6 well plates with either 5 μM scrambled control siRNA (Dharmacon, D-001810-10-05), or 5 µM ON-TARGETplus siRNA against *Igf1r* (Dharmacon, L-056843-00-0005) or against *Insr* (Dharmacon, L-043748-00-0005), or a combination of both *Igf1r* and *Insr* (double knockdown). Transfection was achieved using 3.5 µl of DharmaFECT 2 transfection reagent (Dharmacon, T-2002-01) per well according to manufacturer’s instructions.

### 2.9 *In vitro* T cell activation assay

Primary murine splenocytes were isolated from spleens of C57BL/6 mice as described previously (6). Dissected spleens were dissociated in MAC buffer and passed through a 70 μm cell strainer to obtain a single-cell suspension. Cells were centrifuged (300 × g), and red blood cells were lysed using 1× Red blood lysis buffer (Biolegend). Obtained splenocytes were cultured in either primary pancreatic fibroblast conditioned media in which fibroblasts had been treated with either IgG control antibody (100 µg/ml) or IGF-blocking antibody (100 µg/ml). As a positive and negative control separate splenocytes were cultured in serum free RPMI 1640 medium with or without Dynabeads Mouse T activator CD3/CD28 (Thermofisher, 11452D). Cells were plated in 96-well plates and incubated at 37°C for 24 hours. Subsequently, Brefeldin A (eBioscience; 1:100) was added to the cells for 5 hours. Cells were then harvested, stained with LIVE/DEAD™ Fixable Aqua Stain (1:1000, Invitrogen, L34957 before subsequent fixation and permeabilisation using FIX & PERM™ Cell Permeabilization Kit (Invitrogen). Fixed cells were then incubated with conjugated antibodies against anti-CD8-PerCP/Cyanine5.5 (1:100 Biolegend; clone 53-6.7) and IFNγ-PE (1:100, Biolegend; clone XMG1.2) for 45 minutes prior to flow cytometric analysis on a FACS Canto II (BD bioscience) instrument.

### 2.10 Human studies

Human studies using primary tissue samples were approved by the National Research Ethics (NRES) Service Committee North West – Greater Manchester REC15/NW/0477 and REC19/NW/0298. All samples included in the analysis were histologically confirmed as PDAC by an independent team of histopathologists at the Royal Liverpool University Hospital NHS Trust. All individuals provided informed consent for tissue donations on approved institutional protocols.

### 2.11 Precision cut tumour slicing and *ex vivo* culture

Precision cut tumour slicing (PCTS) was performed on fresh primary PDAC specimens following a protocol adapted from (7, 41, 42). Briefly, 0.5 cm samples of primary PDAC tissues were embedded in 3% UltraPureLMP Agarose (Invitrogen) dissolved in PBS, onto specimen small dishes. Sectioning was performed using Leica vibrating blade microtome VT1200 S (Leica), using stainless steel razor blades (Personal Medical) under buffered conditions with ice-cold HBSS containing 25 mM glucose (Merck), at the following adjustable settings: knife angle: 15°; sectioning speed: 0.4–1 mm/s; oscillation amplitude: 3 mm; step size: 250 µm; retract: 10 µm; continuous stroke. The first slice was immediately fixed in formalin as a day 0 and embedded in paraffin. Slices were cultured on inserts (0.4 µm pore size, 30 mm diameter, Millicell^®^, Millipore, PICM0RG50) placed inside 6 well plates containing 1.1 ml DMEM, high glucose, GlutaMAX (Gibco, 10569010) with 10% FBS and 1% Penicillin/Streptomycin with an additional 150 µl of media being added on top of slices. Inserts were coated with 250 µl of collagen gel consisting of 3 mg/ml rat tail collagen type 1 (Merck, C3867-1VL), 0.025 N NaOH in PBS. Slices were cultured for 24 hours at 37°C, 5% CO2 before media was replaced and slices were treated with either IgG control antibody (100 µg/ml) or IGF-blocking antibody (100 µg/ml) for a further 72 hours of culture at the same conditions. Treated slices were fixed in formalin and embedded in paraffin prior to downstream immunohistochemical and immunofluorescent analysis. Conditioned media from treated slices was filtered at 0.22 µm and stored at -20°C until use.

### 2.12 Chemotaxis assays

In the mouse model experiment primary murine CD8^+^ T cells were obtained from the spleen of C57BL/6 mice using the murine CD8a^+^ T Cell Isolation Kit (Miltenyi, 130-104-075) according to manufacturer’s instructions. 1 x 10^6^ CD8^+^ T cells were added in serum free RPMI into 5 µm transwell inserts (Corning) and allowed to migrate into the bottom chamber of the 24- well plate for 15 hours. The bottom chamber was loaded with fibroblast conditioned media whereby primary murine fibroblasts had been cultured in fibroblast conditioned media treated with either IgG control antibody (100 µg/ml) or IGF-blocking antibody (100 µg/ml) for 48 hours. CD8^+^ T cells were treated with or without the CXCR3 antagonist SCH 546738 (10 nM, MedChemExpress), normal goat IgG control (1 µg/ml, R&D Systems, AB-108-C) or Human/Mouse IGF-I R/IGF1R Antibody (1 µg/ml, R&D Systems, AF-305-SP) and their migration towards anti-IGF treated fibroblast conditioned media measured after 15 hours. As a positive and negative control, the bottom chamber was loaded with serum free RPMI +/- recombinant murine CXCL9 (1000 ng/ml, Peprotech).

In the human model experiment, 1 x 10^6^ Jurkat cells were added in DMEM, high glucose, GlutaMAX media into upper chamber of 5 µm inserts and their migration towards conditioned media generated from PCTS *ex vivo* human PDAC slices treated with either IgG control antibody (100 µg/ml) or IGF-blocking antibody (100 µg/ml) measured after 15 hours. For both experiments and all treatments, both the top and bottom chamber was supplemented with recombinant murine IL-2 (50 U/ml, Peprotech). Migrated cells were recovered from the lower chamber and counted by using a haemocytometer.

### 2.13 RT-qPCR

Total RNA purification was performed with the RNeasy Kit (Qiagen), and cDNA was generated using the M-MLV Reverse Transcriptase kit (Invitrogen). 500 ng of total RNA was used to generate cDNA. qPCR was performed using 5 x HOT FIREPol EvaGreen qPCR Mix Plus (ROX; Solis Biodyne) on an AriaMx Real-Time PCR (qPCR) Instrument (Agilent). Three-step amplification was performed (95°C for 15 seconds, 60°C for 20 seconds, and 72°C for 30 seconds) for 45 cycles. Relative expression levels were normalized to Gapdh expression according to the formula: 2^^-(Ct gene of interest – Ct Gapdh)^. Fold increase in expression levels was calculated by the comparative Ct method: 2^^(-ddCt).^ The control used to apply 2^^(-ddCt)^ was either the IgG control antibody treatment or the scrambled siRNA control as applicable. The following QuantiTect Primers Assays (Qiagen) were used to assess mRNA levels: Mm-Gapdh (Mm_Gapdh_3_SG; QT01658692), Mm-IL10 (Mm_ IL10_1_SG; QT00106169), Mm-Tgfb1 (Mm_Tgfb1_1_SG; QT00145250), Mm-IL6 (Mm_Il6_1_SG QuantiTect; QT00098875), Mm-Tnf (Mm_Tnf_1_SG; QT00104006), Mm-Cxcl10 (Mm_Cxcl10_1_SG; QT00093436), Mm-Cxcl12 (Mm_Cxcl12_va.1_SG), Mm-Arginase (Mm_Arg1_1_SG; QT00134288), Mm-Col1a1 (Mm_Col1a1_1_SG; QT00162204), Mm-Col1a2 (Mm_Col1a2_1_SG; QT01055572), Mm-Fn1 (Mm-Fn1_1_SG; QT00135758), Mm-Stat1 (Mm-Stat1_1_SG; QT00162183), Mm-Insr (Mm-Insr_vb.1_SG; QT01540854), Mm-Igf1r (Mm-Igf1r_1_SG; QT00155351). Mm-Cxcl9 (Mm-Cxcl9_Fw CAGCTCTGCCATGAAGTCCG; Mm-Cxcl9_Rev TCCTTATCACTAGGGTTCCTCG), Mm-Icam1 (Mm-Icam1_Fw GAGCTCGAGAGTGGACCCAA; Mm-Icam1_Rev TCTCAGCTCCACACTCTCCG), Mm-Irf1 (Mm-Irf1_Fw CGGGCATCTTTCGCTTCGT; Mm-Irf1_Rev AGGGTCTCATCCGCATTCGAG), Mm-Oas2 (Mm-Oas2_Fw CAGGAGGGATCTTGTGGCAGG; Mm_Oas2_Rev TGCCAGATCACTCCAGAAGCG) were purchased from Merck.

### 2.14 Immunohistochemistry analysis

Deparaffinization and antigen retrieval were performed using an automated DAKO PT-link. Paraffin-embedded human and mouse PDAC tissues were immunostained using the DAKO envision system-HRP. 4 µm tissue sections were incubated overnight at 4°C with the following primary antibodies: CD3 (Abcam, SP7 clone, ab16669, 1:100, high pH antigen retrieval); CD4 (Abcam, ab183685, 1:500, high pH antigen retrieval); αSMA (Abcam, ab5694, 1:200 high pH antigen retrieval), CXCL10 (Invitrogen, 10H11L3 clone, 701225, 1:100, low pH antigen retrieval); Ki67 (Abcam, ab15580, 1:1000, low pH antigen retrieval); CC3 (Cell Signaling Technology, #9661, 1:200, high pH antigen retrieval); phospho-Insulin/IGF1 receptors (R&D, AF2507, 1:50 high pH antigen retrieval). Secondary HRP-conjugated antibodies were incubated for 30 minutes at room temperature. Staining was developed using diaminobenzidine and counterstained with haematoxylin.

### 2.15 Immunofluorescent analysis

For immunofluorescence staining, 4 µm tissue sections were permeabilised by 0.1% TritonX-100 (Sigma Aldrich) for 2 min at room temperature. Unspecific bindings were prevented by using PBS + 10% donkey serum for 1 hour at room temperature. Tissue sections were incubated overnight at 4°C with the following antibodies: CD8a (eBioscience, 53-6.7 clone, 14-0081-82, 1:50); CD8 (Dako, C8/144B clone, M7103, 1:100), Granzyme B (Abcam, ab4059, 1:100); PD-1 (Abcam, ab214421, 1:500); Ki67 (Abcam, ab15580, 1:1000); CC3 (Cell Signaling Technology, #9661, 1:200); CD4 (Invitrogen, 14-9766-80, 4SM95 clone, 1:50); FoxP3 (Cell Signaling Technology, #12653, 1:100); F4/80 (Biolegend, BM8 clone, 123101 1:50); F4/80 (Cell Signaling Technology, #70076, 1:100), CD206 (Abcam, ab64693, 1:1000); MHC II (Novus Biologicals, NBP1-43312, 1:100); CXCL9 (R&D Systems, AF-492-NA, 1:50); phospho-Stat3 (Tyr705) (Cell Signaling Technology, #4093, 1:100); phospho-Stat1 (Tyr701) (Cell Signaling Technology, #9167, 1:300); PDGFRβ (R&D Systems, AF1042, 1:50); αSMA (Abcam, ab5694, 1:200). The following day, sections were washed with PBS and incubated with 5 mg/ml 4′,6-diamidino-2-phenylindole (DAPI) and fluorescently labelled secondary antibodies for 2 hrs at room temperature: Donkey anti rat AF488 (Abcam, ab150149, 1:300); Donkey anti-rat AF647 (Abcam, ab150155, 1:300); Donkey anti-rabbit AF488 (Biolegend, 406416, 1:300); Donkey anti-rabbit AF594 (Biolegend, 406418 1:300); Donkey anti-goat AF488 (Abcam, ab150129, 1:300); Donkey anti-goat AF594 (Abcam, ab150132, 1:300); Donkey anti-mouse AF488 (Abcam, ab150105, 1:300). Sections were mounted onto coverslips using Dako Fluorescent mounting medium (Agilent). Slides were imaged using Axio Observer Light Microscope with the Apotome.2 (Zeiss). Positive cells were counted manually (using 5-10 field of view per sample) whereas cell nuclei counting was automated using QuPath (v0.2.3).

### 2.16 Immunocytochemistry analysis

Primary pancreatic fibroblasts were grown on cover slips and fixed with 4% paraformaldehyde in PBS (VWR, ALFAJ19943.K2) for 10 min at room temperature with gentle agitation. Cells were washed gently with PBS and permeabilised by 0.1% TritonX-100 (Sigma Aldrich) for 2 min at room temperature. Cells were blocked in 2% BSA/PBS for 30 min at room temperature with gentle agitation before subsequent incubation overnight at 4°C with primary antibody against anti-STAT1 (Cell Signaling Technology, #9172, 1:100). The following day, cells were washed with PBS and incubated with 5mg/ml 4′,6-diamidino-2-phenylindole (DAPI) and Donkey anti-rabbit AF594 (Biolegend, 406418 1:300) for 2 hr room temperature. Following incubation cells were washed with PBS and mounted onto microscope slides with Dako Fluorescent mounting medium (Agilent). Slides were imaged using Axio Observer Light Microscope with the Apotome.2 (Zeiss) with the total STAT1^+^ and total cell nuclei quantified using QuPath (v0.2.3) software.

### 2.17 Immunoblotting

Primary pancreatic fibroblasts or primary BMDMs were lysed in 62.5 mM Tris-HCl pH 6.8, 10% glycerol, 2% SDS, 1% β-mercaptoethanol) supplemented with complete protease inhibitor mixture (Sigma), phosphatase inhibitor cocktail (Invitrogen), 1 mM phenylmethylsulfonyl fluoride and 0.2 mM sodium orthovanadate. Following sonication and clarification, protein concentration was determined using Pierce Protein BCA Assay Kit – Reducing Agent Compatible (Thermo Fisher) according to manufacturer’s instructions. 30 µg of cell lysates were loaded and ran on 10% SDS-PAGE gels. Conditioned media generated from PCTS *ex vivo* human PDAC slices was concentrated using SrataClean Resin (Agilent), loaded and ran on 15% SDS-PAGE gels. After protein transfer using the Trans-blot Turbo Transfer System (Bio-rad), PVDF membranes were blocked in 5% BSA-TBST for 1 hr and blotted overnight at 4 ˚C with the following primary antibodies: anti-IGF1R (R&D Systems, AF305-NA, 1:1000); anti-Insulin receptor (Abcam, ab137747,1:1000); anti-phospho-AKT (Ser473) (Cell Signaling Technology, #4060, 1:1000); anti-AKT (Cell Signaling Technology, #9272, 1:1000); anti-phospho-Stat3 (Tyr705) (Cell Signaling Technology, #4093, 1:1000); anti-STAT3 (Cell Signaling Technology, #4094, 1:1000), anti-GAPDH (Sigma, G9545, 1:10,000); anti-CXCL9 (R&D Systems, AF392, 1:2000), anti-tubulin (Sigma, T6199, 1:10,000); followed by HRP-conjugated secondary antibodies for 2 hours at room temperature. Protein bands were visualised using Pierce ECL Western Blotting Substrate (Thermo Fisher) on a ChemiDoc MP (Bio-rad) imaging system.

### 2.18 Picrosirius red staining

Paraffin-embedded human and mouse PDAC samples were dewaxed and hydrated using a graded ethanol series. Tissue sections were then treated with 0.2% phosphomolybdic acid and subsequently stained with 0.1% Sirius Red F3B (Direct Red 80; Sigma Aldrich) in saturated picric acid solution for 90 minutes at room temperature. Tissues were then rinsed twice in acidified water (0.5% glacial acetic acid; Sigma Aldrich) before and after the staining with 0.033% fast green FCF (Sigma Aldrich). Finally, tissues were dehydrated in three changes of 100% ethanol, cleared in xylene, and mounted. Picrosirius red staining was quantified using Image J software.

Quantification of collagen deposition by primary pancreatic fibroblasts was measured following a protocol adapted from (43). Following 72 hours of culture with IgG control antibody (100 µg/ml) or IGF-blocking antibody (100 µg/ml), primary pancreatic fibroblasts were fixed *in situ* with 70% ice cold ethanol and transferred to -80°C for 30 min. Cells were subsequently stained with 0.1% Sirius Red F3B (Direct Red 80; Sigma Aldrich) in saturated picric acid solution and incubated overnight at 4°C with gentle agitation. Unbound dye was washed away with distilled water and fixed cells were subsequently treated with 1 M NaOH at room temperature for 10 min with gentle agitation. 100 µl of dissolved collagen-dye complex was transferred in duplicate to a 96 well microplate and absorbance measured at 490 nm on a Varioskan Flash Spectral Scanning Multimode Reader (Thermo Fisher Scientific). A standard curve was constructed by drying known concentrations of rat-tail collagen type I (Merck, C3867-1VL), on the surface of tissue culture plastic before staining, dissolving and quantification as described above.

### 2.19 Statistical analysis

Statistical significance (analysed with GraphPad Prism v8 software) was determined using two-tailed unpaired Student’s t tests when comparing differences between two experimental groups for parametric data or Mann-Whitney U test for non-parametric data. Unless otherwise stated, one-way ANOVA with Dunnett’s multiple comparisons test was used for all experiments with more than two groups. For **Figures 4J** and **4K** two-way ANOVA with Dunnett’s multiple comparisons test was performed. For **Figure 5B** and **Supplementary Figure 5A** one sample t test with a theoretical mean of 1 was performed. For **Figure 5G** and **Supplementary Figure 1F** two-way ANOVA with Bonferroni’s multiple comparisons test was performed. A P-value < 0.05 was considered statistically significant and P values are indicated in the figures using asterisks: *P<0.05; **P<0.01; ***P<0.001; ****P<0.0001; ns denotes not significant.

## 3 Results

### 3.1 IGF blockade leads to an increase in cytotoxic T cell accumulation in pancreatic tumours.

**Figure 1:**
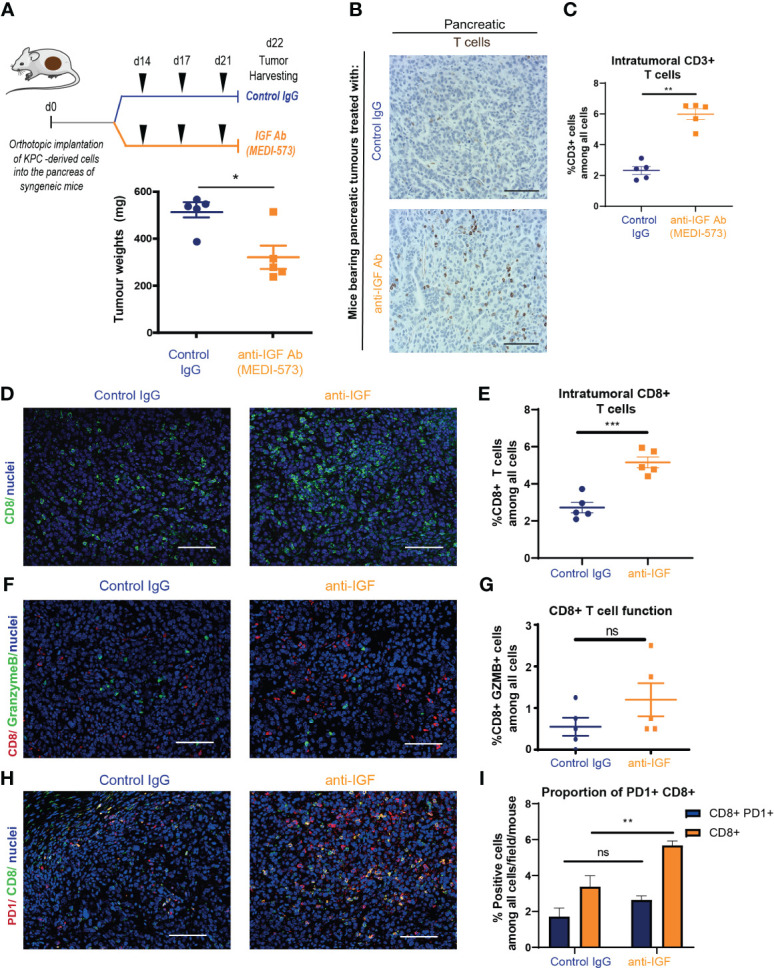
IGF blockade leads to an increase in cytotoxic T cell accumulation in pancreatic tumours **(A)** Top, *LSL-Kras^G12D/+^;LSL-Trp53^R172H/+^;Pdx-1-Cre* (KPC) derived FC1242 cells were orthotopically implanted into the pancreas tail of syngeneic C57BL-6J recipient mice. Mice were treated by intraperitoneal injection with either IgG2 control antibody (60 mg/kg) or IGF-blocking antibody MEDI-573 (60 mg/kg) at days 14, 17 and 21 post implantation. Pancreatic tumours were harvested at day 22 post implantation. Below, tumour weights at harvest for both treatment groups (n = 5 mice per treatment group), *P ≤ 0.05 using Mann-Whitney U test. **(B)** Immunohistochemical staining of CD3^+^ T cells in formalin fixed paraffin embedded tissues from orthotopic murine PDAC tumours treated with IgG2 control antibody or IGF-blocking antibody MEDI-573. Scale bar; 50 μm. **(C)** Quantification of CD3 staining. Data displayed as total CD3+ T cells among all cells. A total of 5-8 fields of view counted/mouse tumour, n = 5 mice per treatment group, *P ≤ 0.05 using Mann-Whitney U test. **(D)** Immunofluorescent staining of CD8 (green), and nuclei (blue) in formalin fixed paraffin embedded tissues from orthotopic murine PDAC tumours treated with IgG2 (control) antibody or IGF blocking antibody MEDI-573. Scale bar 50 μm. **(E)** Quantification of CD8 staining. Data displayed as total CD8^+^ T cells among all cells. A total of 5-8 fields of view counted/ mouse tumour, n = 5 mice per treatment group, ***P ≤ 0.001 using unpaired t test. **(F)** Immunofluorescent staining of CD8 (red), granzyme B (green) and nuclei (blue) in formalin fixed paraffin embedded tissues from orthotopic murine PDAC tumours treated with IgG2 (control) antibody or IGF blocking antibody MEDI-573. Scale bar 50 μm. **(G)** Quantification of functionally active CD8^+^ T cells in IgG control treated and anti-IGF treated orthotopic murine pancreatic tumours. Data displayed as percentage of CD8^+^/GranzymeB^+^ cells among all cells. A total of 5-8 fields of view counted/mouse tumour, n= 5 mice per treatment group, ns; P > 0.05 using Mann-Whitney U test. **(H)** Immunofluorescent staining of CD8 (green), PD1 (red) and nuclei (blue) in formalin fixed paraffin embedded tissues from orthotopic murine PDAC tumours treated with IgG2 (control) antibody or IGF blocking antibody MEDI-573. Scale bar 50 μm. **(I)** Quantification of PD1^+^ CD8^+^ T cells in IgG control treated and anti-IGF treated orthotopic murine pancreatic tumours. Data displayed as either percentage of CD8^+^ T cells among all cells or PD1^+^/CD8^+^ T cells among all cells. A total of 5-8 fields of view counted/mouse tumour, n= 5 mice per treatment group, ns; P > 0.05, **P ≤ 0.01 using Mann-Whitney U test.

Mice bearing established orthotopic PDAC tumours were treated with either IgG2 control antibody or IGF-blocking antibody (MEDI-573). In line with previous work in our lab and others (3, 44), treatment with IGF-blocking antibody alone only led to a modest decrease in tumour weight (**Figure 1A**). However, further analysis of these tumours through immunohistochemistry revealed a significant increase in the number of tumour infiltrating lymphocytes (TILs) upon IGF blockade as determined by the percentage of CD3^+^ T cells (**Figures 1B, C**). Further characterisation of these TILs by immunohistochemistry and immunofluorescent analysis revealed no significant difference in the number of CD4^+^ T cells (**Supplementary Figures S1A, B**) but a significant difference in the number of CD8^+^ cytotoxic T cells upon IGF blockade (**Figures 1D, E**). Of note, the number of regulatory T cells (Tregs; CD4^+^ FoxP3^+^) remained unchanged by IGF inhibition (**Supplementary Figures S1C, D**). Corroborating these results, the overall increase in CD8^+^ T cells in tumours treated with IGF-blocking antibody was also observed in whole tumour digests using CyTOF mass cytometry (**Supplementary Figures S1E, F**). Despite the overall increase in CD8^+^ T cells within PDAC tumours upon IGF blockade, the proportion of CD8^+^ T cells concomitantly expressing granzyme B, a marker of T cell functional activity, remains low after IGF blockade (**Figures 1F, G**). Additionally, the fraction of CD8^+^ T cells which are positive for the inhibitory immune checkpoint PD-1 and the activation marker CD69 is unaffected by IGF blockade (**Figures 1H, I**; **Supplementary Figures S1E, F**), suggesting that while their overall numbers increase, these CD8^+^ T cells remain functionally inactive (45). Corroborating this, the proportion of CD8^+^ PD1^+^ T cells among all CD8^+^ T cells remains unaffected by IGF blockade (**Supplementary Figure S1G**).

Taken together, these data show that IGF blockade leads to an increase in CD8^+^ accumulation within primary pancreatic tumours. Despite this overall increase in CD8^+^ T cells within the tumour, the resulting decrease in tumour size upon treatment with anti-IGF antibody is modest, which can be explained at least in part by the fact that these T cells remain functionally inactive.

### 3.2 IGF blockade does not affect CD8^+^ T cell priming, survival or proliferation within the PDAC TME

The cancer immunity (CI) cycle proposed by Chen and Mellman (and recently updated (46) describes the series of stepwise events necessary for an effective anti-tumour immune response, beginning with the release of cancer neoantigens and culminating with the targeted CD8^+^ cytotoxic T cell mediated destruction of tumour cells. In order for CD8^+^ T cells to accumulate within the tumour, they must first be primed/activated in the tumour draining lymph node. Following this they must effectively traffic towards the tumour bed, infiltrate into the tumour and stroma, and survive within the hypoxic, immunosuppressive conditions of the TME maintaining their effector states and function (46). We therefore hypothesised that aberrant IGF signalling affects one/or multiple steps in this cycle to impede T cell accumulation within the pancreatic TME.

**Figure 2:**
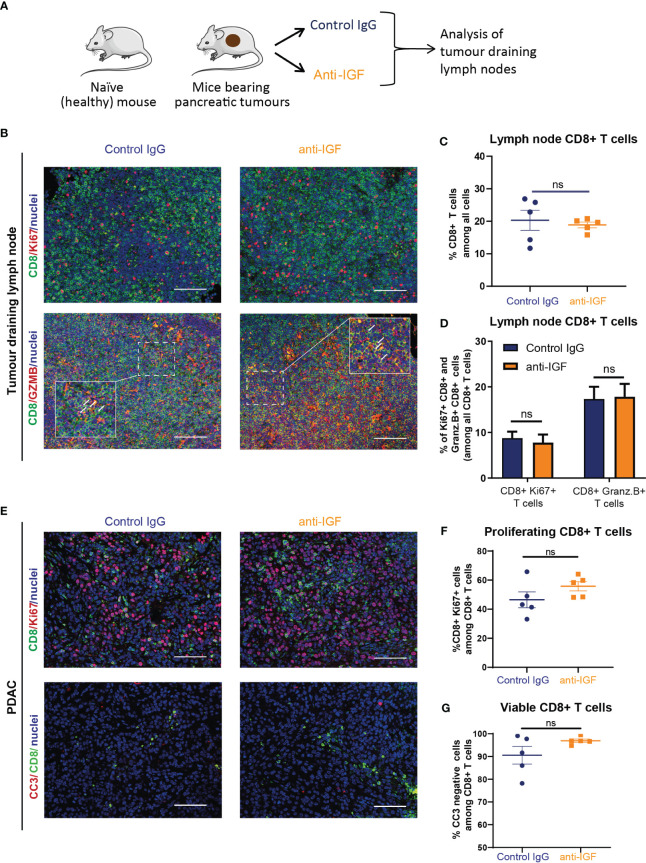
IGF blockade does not affect CD8^+^ T cell priming, survival or proliferation within the PDAC TME. **(A)** *LSL-Kras^G12D/+^;LSL-Trp53^R172H/+^;Pdx-1-Cre* (KPC) derived FC1242 cells were orthotopically implanted into the pancreas tail of syngeneic C57BL-6J recipient mice. Mice were treated by intraperitoneal injection with either IgG2 control antibody (60 mg/kg) or IGF-blocking antibody MEDI-573 (60 mg/kg) at days 14, 17 and 21 post implantation. Mesenteric lymph nodes were harvested at day 22 post implantation. **(B)** Top, immunofluorescent staining of CD8 (green), Ki67 (red) and nuclei (blue) in formalin fixed paraffin embedded mesenteric lymph nodes from mice bearing orthotopic PDAC tumours treated with IgG2 (control) antibody or IGF blocking antibody MEDI-573. Scale bar 50 μm. Below, immunofluorescent staining of CD8 (green), Granzyme B (red) and nuclei (blue) in formalin fixed paraffin embedded mesenteric lymph nodes from mice bearing orthotopic PDAC tumours treated with IgG2 (control) antibody or IGF blocking antibody MEDI-573. Scale bar 50 μm. **(C)** Quantification of CD8 staining in mesenteric lymph nodes. Data displayed as total CD8^+^ T cells among all cells. A total of 3 fields of view counted/mouse lymph node, n = 5 mice per treatment group, ns; P > 0.05 using Mann-Whitney U test. **(D)** Quantification of Ki67+/CD8+ T cells and Granzyme B+/CD8+ T cells in mesenteric lymph nodes in IgG control treated and anti-IGF treated mice bearing orthotopic pancreatic tumours. Data displayed as percentage of Ki67^+^/CD8^+^ T cells and Granzyme B^+^/CD8^+^ T cells among all CD8^+^ T cells. A total of 3 fields of view counted/mouse tumour, n= 5 mice per treatment group, ns; P > 0.05 using Mann-Whitney U test. **(E)** Top, immunofluorescent staining of CD8 (green), Ki67 (red) and nuclei (blue) in formalin fixed paraffin embedded tissues from orthotopic murine PDAC tumours treated with IgG2 (control) antibody or IGF blocking antibody MEDI-573. Bottom, Immunofluorescent staining of CD8 (green), cleaved caspase 3 (red) and nuclei (blue) in formalin fixed paraffin embedded tissues from orthotopic murine PDAC tumours treated with IgG2 (control) antibody or IGF blocking antibody MEDI-573. Scale bar 50 μm. **(F)** Quantification of proliferating CD8^+^ T cells in IgG control treated and anti-IGF treated orthotopic murine pancreatic tumours. Data displayed as percentage of CD8^+^/Ki67^+^ cells among all CD8+ T cells. **(G)** Quantification of viable CD8^+^ T cells in IgG control treated and anti-IGF treated orthotopic murine pancreatic tumours. Data displayed as percentage of CD8^+^/CC3- cells among all CD8^+^ T cells. A total of 5-8 fields of view counted/mouse tumour, n= 5 mice per treatment group, ns; P > 0.05 using Mann-Whitney U test.

Immunofluorescent analysis of tumour draining mesenteric lymph nodes from mice bearing orthotopic PDAC tumours (**Figure 2A**) revealed no differences in the numbers of CD8^+^ T cells upon treatment with IGF blocking antibody (**Figures 2B, C**). In addition, no differences in the proportion of proliferative (Ki67^+^) or functionally active (Granzyme B^+^) CD8^+^ T cells were observed, suggesting that IGF blockade is unlikely to affect T cell priming (**Figures 2B, D**). Following this we sought to further characterise the CD8^+^ T cells present within primary PDAC tumours following anti-IGF treatment. Again, no differences in CD8^+^ T cell proliferation were detected (**Figures 2E, F**) and additionally, no differences in their survival (cleaved caspase 3^+^) (**Figures 2E, G**) were observed upon IGF blockade.

Collectively, these data show that IGF blockade is unlikely to affect CD8^+^ T cells priming, does not affect CD8^+^ T cell survival or proliferation within the PDAC TME and that their increased accumulation in tumours must be attributed to other factors affecting different steps of the CI cycle.

### 3.3 IGF blockade promotes the production of T cell chemoattractants CXCL9 and CXCL10 by TAMs and CAFs

**Figure 3:**
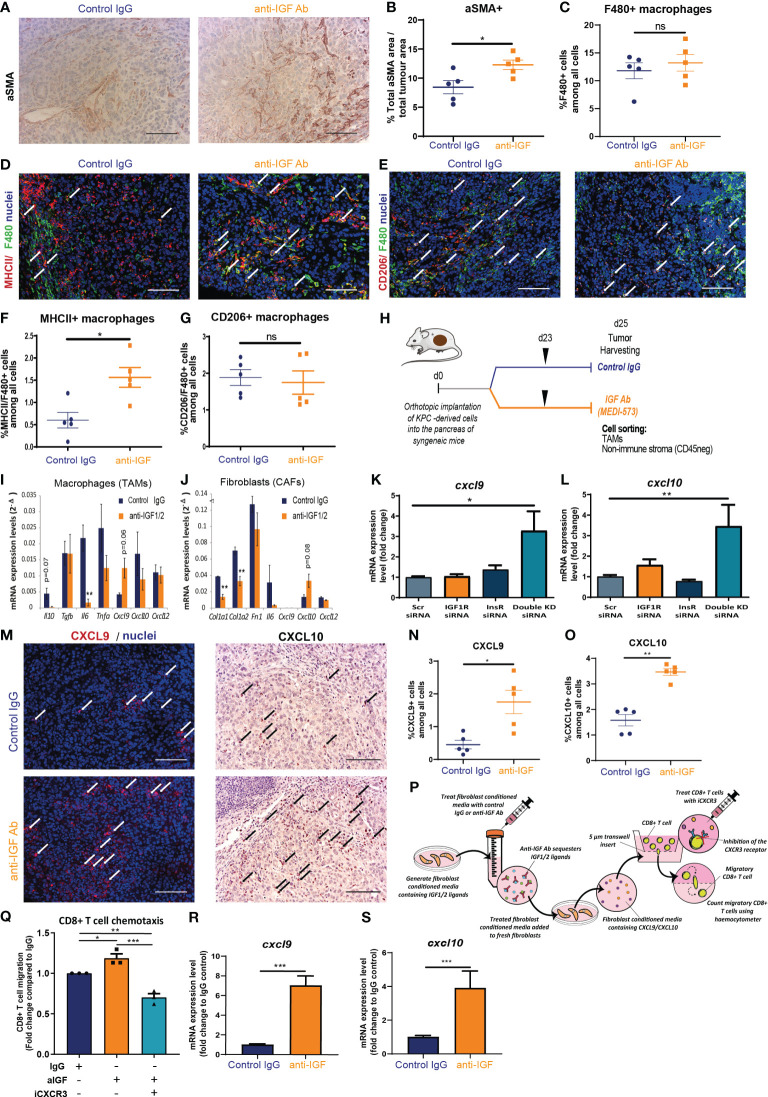
TAM and CAF derived chemokines CXCL9 and CXCL10 facilitate CD8^+^ T cell recruitment to PDAC tumours upon IGF blockade. **(A)** Immunohistochemical staining of αSMA in formalin fixed paraffin embedded tissues from orthotopic murine PDAC tumours treated with IgG2 control antibody or IGF-blocking antibody MEDI-573. Scale bar; 50 µm. **(B)** Quantification of αSMA staining. Data displayed as total αSMA^+^ area/total tumour area. A total of 5-8 fields of view counted/mouse tumour, n = 5 mice per treatment group, *P ≤ 0.05 using Mann-Whitney U test. **(C)** Quantification of F480 staining. Data displayed as % F480^+^ cells among all cells. A total of 5-8 fields of view counted/mouse tumour, n = 5 mice per treatment group, ns; P > 0.05 using Mann-Whitney U test. **(D)** Immunofluorescent staining of F480 (green), MHCII (red) and nuclei (blue) in formalin fixed paraffin embedded tissues from orthotopic murine PDAC tumours treated with IgG2 (control) antibody or IGF blocking antibody MEDI-573. Scale bar 50 µm. White arrows denote cells which are positive for both F480 and MHCII. **(E)** Immunofluorescent staining of F480 (green), CD206 (red) and nuclei (blue) in formalin fixed paraffin embedded tissues from orthotopic murine PDAC tumours treated with IgG2 (control) antibody or IGF blocking antibody MEDI-573. Scale bar 50 µm. White arrows denote cells which are positive for both F480 and CD206. **(F)** Quantification of MHCII^+^/F480^+^ macrophages in IgG control treated and anti-IGF treated orthotopic murine pancreatic tumours. Data displayed as percentage of MHCII^+^/F480^+^ cells among all cells. A total of 5-8 fields of view counted/mouse tumour, n= 5 mice per treatment group, * P ≤ 0.05 using Mann-Whitney U test. **(G)** Quantification of CD206^+^/F480^+^ macrophages in IgG control treated and anti-IGF treated orthotopic murine pancreatic tumours. Data displayed as percentage of CD206^+^/F480^+^ cells among all cells. A total of 5-8 fields of view counted/mouse tumour, n= 5 mice per treatment group, ns; P > 0.05 using Mann-Whitney U test. **(H)** *LSL-Kras^G12D/+;^LSL-Trp53^R172H/+;^Pdx-1-Cre* (KPC) derived FC1242 cells were orthotopically implanted into the pancreas of syngeneic recipient (C57BL/6J) mice. Mice were treated with IgG2 control antibody or IGF-blocking antibody MEDI-573 at days 23. Tumours were harvested and digested at day 25 post implantation with TAMs (CD45^+^/F4/80^+^ cells) and non-immune stromal cells (CD45^-^/zsGreen^-a^) being sorted by flow cytometry and subsequently subjected to transcriptional analysis. **(I)** Quantification of *Il10, Tgfb, IL6, Tnfa, Cxcl9, Cxcl10* AND *Cxcl12* mRNA expression levels in zsGreen^-^/CD45^+^/F4/80^+^ tumour associated macrophages isolated from murine PDAC tumours treated with IgG2 control antibody or IGF-blocking antibody MEDI-573 (n=3). **P ≤ 0.01 using unpaired t tests **(J)** Quantification of *Col1a1, Col1a2, Fn1, Il6, Cxcl9, Cxcl10* and *Cxcl12* mRNA expression levels in zsGreen^-^/CD45^-^ stromal fibroblasts isolated from murine PDAC tumours treated with IgG2 control antibody or IGF-blocking antibody MEDI-573 (n=3). **P ≤ 0.01 using unpaired t tests. **(K)** Quantification of *Cxcl9* and **(L)** *Cxcl10* mRNA expression levels in primary murine fibroblasts isolated from the pancreata of wild-type C57BL/6J mice and treated with scrambled control siRNA (5 µM) *Igfr1* siRNA (5 µM) *Igf1r* (5 µM) or a combination of both *Igf1r* and *Insr* siRNAs (5 µM). Expression data displayed as fold change compared to scrambled control siRNA treatment *, *P* ≤ 0.05; **P ≤ 0.01 using one-way ANOVA with Tukey’s multiple comparisons *post hoc* test. **(M)** Left, Immunofluorescent staining of CXCL9 (red) and nuclei (blue) in formalin fixed paraffin embedded tissues from orthotopic murine PDAC tumours treated with IgG2 (control) antibody or IGF blocking antibody MEDI-573. White arrows denote CXCL9^+^ cells. Right, Immunohistochemical staining of CXCL10 in formalin fixed paraffin embedded tissues from orthotopic murine PDAC tumours treated with IgG2 control antibody or IGF-blocking antibody MEDI-573. Black arrows denote CXCL10^+^ cells. Scale bar 50 µm. **(N)** Quantification of CXCL9^+^ cells in IgG control treated and anti-IGF treated orthotopic murine pancreatic tumours. Data displayed as percentage of CXCL9^+^ cells among all cells. A total of 5-8 fields of view counted/mouse tumour, n= 5 mice per treatment group, *P ≤ 0.05 using Mann-Whitney U test. **(O)** Quantification of CXCL10^+^ cells in IgG control treated and anti-IGF treated orthotopic murine pancreatic tumours. Data displayed as percentage of CXCL10^+^ cells among all cells. A total of 5-8 fields of view counted/mouse tumour, n= 5 mice per treatment group, **P ≤ 0.01 using Mann-Whitney U test. **(P)** Summary schematic for CD8^+^ T cell chemotaxis assay. Fibroblast conditioned media was generated from primary murine fibroblasts isolated from the pancreata of wild-type C57BL/6J mice and treated with IgG control antibody (100 µg/ml) or IGF-blocking antibody MEDI-573 (100 µg/ml). Fresh primary murine fibroblasts were cultured in treated fibroblast conditioned media for 48 hours and conditioned media collected for use in CD8^+^ T cell chemotaxis assays. Migration of primary murine CD8^+^ T cells through a 5 µm transwell insert towards fibroblast conditioned media was measured after 15 hours using a haemocytometer. CD8^+^ T cells were treated with or without the CXCR3 antagonist SCH 546738 (10 nM) before inclusion in migration assay. **(Q)** Data are presented as the number of migratory T cells as a fold change compared to the IgG control treated fibroblast conditioned media AFTER 15 hr. n=3, *P ≤ 0.05, **P ≤ 0.01, ***P ≤ 0.001 using one-way ANOVA with Tukey’s multiple comparisons test. **(R)** Quantification of *Cxcl9* and **(S)** *Cxcl10* mRNA expression levels in primary murine fibroblasts isolated from the pancreata of wild-type C57BL/6J mice and treated with IgG control antibody (100 µg/ml) or IGF-blocking antibody MEDI-573 (100 µg/ml) for 24 hours. Expression data displayed as fold change compared to IgG control treatment. n=3, ***P ≤ 0.001 using Mann-Whitney U test.

TAMs and CAFs remain two of the most abundant non-cancerous cell populations within the PDAC tumour microenvironment and both cell populations play a pleiotropic role in regulating CD8^+^ T cell accumulation within solid tumours via multiple mechanisms (20, 47–49). In addition to this, previously published work from our group demonstrated that TAMs and CAFs are the main extracellular source of IGF ligands within both the pancreatic and TNBC TME (3, 30). In addition, both primary murine macrophages and primary murine fibroblasts express IGF-1R and Insulin receptor (**Supplementary Figures S2A, B**) potentially indicative of an autocrine signalling axis. In accordance, immunohistochemical and immunofluorescent analysis of murine orthotopic PDAC tumours reveals overall changes on TAM and CAF populations when mice are treated with IGF-blocking antibody. Here we demonstrate an increase in αSMA^+^ CAFs upon IGF blockade (**Figures 3A, B**). With regard to TAMs, no changes in total F4/80^+^ TAM numbers are observed (**Figures 3C, D**), yet we demonstrate an increase in MHCII^+^ F480^+^ macrophages, that have been described as a immunostimulatory population of TAMs (50, 51), upon IGF blockade (**Figures 3D, F**). No changes in CD206^+^ TAMs (**Figures 3E, G**) are observed upon IGF blockade.

Given the role of IGF signalling in the TAM and CAF cell populations and the observed changes in these two cell populations upon IGF blockade, as well as the concomitant increases in CD8^+^ T cells within pancreatic tumours, we hypothesised that aberrant IGF signalling in TAMs and/or CAFs negatively regulates the accumulation of CD8^+^ T cells within pancreatic tumours through TAM and/or CAF derived factors.

To test this hypothesis, we isolated both TAMs (CD45^+^/F4/80^+^ cells) and non-immune stromal cells (CD45^-^/zsGreen^-^) using FACS from established orthotopic PDAC tumours that had been treated with either IgG2 control antibody or IGF-blocking antibody (**Figure 3H**). We then analysed the transcriptional expression of a panel of cytokines and chemokines known to regulate T cell survival, function or chemoattraction including: *Il10, Tgfb, Il6*, *Tnfa*, *Cxcl9, Cxcl10* and *Cxcl12* (52–64); and extracellular matrix (ECM) components including: *Col1a1, Col1a2* and *Fn1* in CAFs known to affect T cell infiltration into the tumour milieu (6, 20, 65–67)), in response to treatment with IGF-blocking antibody.

Measuring this panel of transcriptional markers by RT-qPCR revealed significant decreases in TAM expression of *Il6* (**Figure 3I**) upon IGF blockade. Interestingly CAF expression of both *Col1a1* and *Col1a2* is significantly decreased upon treatment with IGF-blocking antibody (**Figure 3J**). Furthermore, borderline significant increases in expression are observed for *Cxcl9* in TAMs (**Figure 3I**) and *Cxcl10* in CAFs (**Figure 3J**) upon IGF blockade. An increase in T cell chemokines could serve to facilitate CD8^+^ T cell accumulation into the PDAC TME through increased trafficking/recruitment towards the tumour.

We then sought to corroborate these findings *in vitro* utilising both primary murine macrophages and primary murine fibroblasts isolated from the bone marrow and pancreata of wild type C57BL/6J mice respectively (**Supplementary Figure S2C**). Any changes observed both *in vitro* and *in vivo* are likely to be the result of a direct mechanistic effect of blocking IGF signalling on the cell types in question as opposed to a potentially indirect change mediated through the multitude of interacting cells within the TME.

No significant changes were observed in transcriptional levels of *Il6* in either BMDMs or fibroblasts upon IGF blockade (**Supplementary Figure S2D, E**). A small but statistically significant increase was observed in *Il10* expression in BMDMs (**Supplementary Figure S2D**), as well as an increase in *Col1a2* expression in fibroblasts upon IGF blockade (**Supplementary Figure S2E**). Interestingly, and in accordance with our *in vivo* findings, we observed a statistically significant increase in the transcriptional expression of both *Cxcl9* and *Cxcl10* in the case of both BMDMs (**Supplementary Figure S2D**) and fibroblasts (**Supplementary Figure S2E**) upon IGF blockade. Supporting this, a statistically significant increase in both *Cxcl9* (**Figure 3K**) and *Cxcl10* (**Figure 3L**) was observed upon genetic ablation of the IGF signalling pathway through simultaneous siRNA knockdown of both *Igf1r* and *Insr* in primary murine fibroblasts. IGF ligands can elicit signal transduction through both the IGF-1R and insulin receptor (68, 69), with the IGF-blocking antibody MEDI-573 inhibiting this signalling pathway through scavenging of IGF1/2 ligands. Therefore, targeted genetic ablation of *Igf1r* and *Insr* serves as a surrogate for our pharmacological inhibition of IGF signalling, with statistically significant knockdowns for both these genes being observed upon siRNA treatment (**Supplementary Figure S2F, G**). Given that pharmacological blockade of IGF signalling elicited a reduction in *Il6 in vivo* which was not recapitulated *in vitro*, this discrepancy led to further analysis of primary murine fibroblasts, revealing no changes in *Il6* transcription upon genetic abrogation of IGF signalling (**Supplementary Figure S2H**).

To our knowledge, this direct increase in CD8^+^ T cell chemokines, *Cxcl9* and *Cxcl10* in response to IGF blockade has not been previously reported in any cell type. Significantly, our results demonstrate that this increase is conserved across both primary murine BMDMs and fibroblasts, and translates into preclinical models of PDAC *in vivo*. Further strengthening these data, immunohistochemical and immunofluorescent analysis revealed an overall significant increase in the levels of CXCL9 and CXCL10 (**Figures 3M–O**) within the TME of mouse PDAC tumours upon IGF blockade.

We next sought to explore whether these observed increases in the T cell chemokines CXCL9 and CXCL10 upon IGF blockade could incur an increase in CD8^+^ T cell migration at a more functional level. To model this, we designed an *in vitro* chemotaxis assay in which the migration of primary murine CD8^+^ T cells towards fibroblast conditioned media was measured, whereby primary murine fibroblasts had been treated with fibroblast conditioned media and either IgG control antibody (100 µg/ml) or IGF-blocking antibody (100 µg/ml) (**Figure 3P**). CD8^+^ T cells preferentially migrated towards fibroblast conditioned media following IGF blockade (**Figure 3Q**) in accordance with transcriptional increases in *Cxcl9* and *Cxcl10* in these fibroblasts (**Figures 3R, S**). In addition to this, pretreatment of CD8^+^ T cells with the CXCR3 antagonist SCH 546738 (10 nM) significantly abrogated their migration towards anti-IGF treated fibroblast conditioned media (**Figure 3Q**). Additionally, blockade of IGF-1R on CD8^+^ T cells had no effect on their migration towards anti-IGF treated fibroblast conditioned media (**Supplementary Figure S2I**), suggesting that the increase in migration is not due to IGF signalling on CD8^+^ T cells themselves. As a positive control we demonstrate a significant increase in CD8^+^ T cell migration towards RPMI upon the addition of recombinant CXCL9 (1000 ng/ml) (**Supplementary Figure S2J**).

Furthermore, flow cytometric analysis of CD8^+^ T cells cultured in fibroblast conditioned media revealed no differences in CD8^+^ T cell viability (CD8^+^ Amycan^-^) or functional status (CD8^+^ IFN-γ^+^) regardless of fibroblast treatment (**Supplementary Figure S3A, B**), supporting our *in vivo* findings that IGF blockade does not affect CD8^+^ survival or function within the PDAC TME. As a positive control we demonstrate a significant increase in CD8^+^ T cell activation upon stimulation with CD3/CD28 Dynabeads (**Supplementary Figures S3C, D**).

Taken together these results highlight a novel role for IGF in regulating the production of the T cell chemokines CXCL9 and CXCL10 by TAMs and CAFs. These data suggest that IGF blockade promotes CD8^+^ T cell accumulation within the PDAC TME at least in part through increased T cell recruitment/trafficking towards the tumour in a stroma-derived CXCL9/CXCL10 chemokine-dependent manner.

### 3.4 IGF blockade reverses phosphorylation of STAT3 in TAMs and CAFs whilst promoting STAT1 transcription of *Cxcl9* and *Cxcl10*

Having demonstrated a clear role of the IGF signalling axis in regulating the expression of the T cell chemokines CXCL9 and CXCL10 in both macrophages and fibroblasts, we investigated this pathway further to gain greater mechanistic insight.

The transcription of *Cxcl9* and *Cxcl10* is a tightly regulated process. Canonically, this occurs under the control of the transcription factor STAT1 following activation of the IFN gamma receptor and subsequent phosphorylation of STAT1 (70–72). It is well established that activation of the IGF signalling pathway promotes the phosphorylation and activation of the downstream effector AKT via the IRS1/2/PI3K/PDK1 cascade (3, 73, 74). Downstream of this, AKT activation promotes the phosphorylation and activation of STAT3 (75), with STAT3 having a pleiotropic role in oncogenesis, via cancer cell intrinsic and extrinsic mechanisms through promotion of an immunosuppressive TME in multiple cancer settings (76–80). Moreover, several studies highlight a role for IGF signalling in promoting immunosuppression via STAT3 activation, regulating this through multiple signalling cascades including JAK1/RACK1/STAT3, as well as the aforementioned PI3K/PDK1/AKT/STAT3 (81–83). Furthermore, AKT phosphorylation can indirectly activate STAT3 via mTOR or PKM2 activation (84–87). Critically, there is mounting evidence to show that STAT3 inhibits the activation and/or nuclear translocation of STAT1 via multiple reported mechanisms (88–91).

We therefore hypothesised that inhibition of the IGF signalling pathway in macrophages and fibroblasts would inhibit the phosphorylation of STAT3. The inhibition of pSTAT3 would in turn attenuate STAT3 repression of STAT1 – ultimately facilitating the transcriptional increases in *Cxcl9* and *Cxcl10* that we observe both *in vitro* and *in vivo* upon IGF blockade (**Figures 3I–O**; **Supplementary Figures S2D, E**).

**Figure 4:**
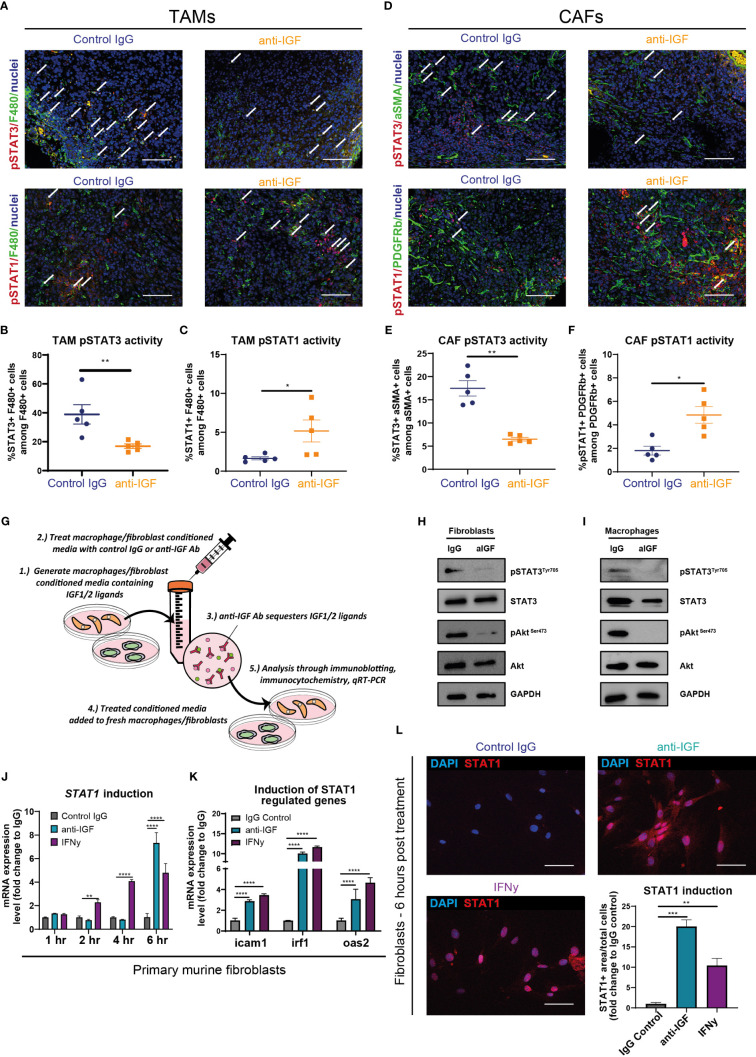
IGF blockade reverses phosphorylation of STAT3 in TAMs and CAFs to facilitate STAT1 induction of *Cxcl9* and *Cxcl10* genes **(A)** Top, immunofluorescent staining of F480 (green), pSTAT3 (red) and nuclei (blue) in formalin fixed paraffin embedded tissues from orthotopic murine PDAC tumours treated with IgG2 (control) antibody or IGF blocking antibody MEDI-573. Bottom, **i**mmunofluorescent staining of F480 (green), pSTAT1(red) and nuclei (blue) in formalin fixed paraffin embedded tissues from orthotopic murine PDAC tumours treated with IgG2 (control) antibody or IGF blocking antibody MEDI-573. Scale bar 50 µm. **(B)** Quantification of the number of F480^+^ macrophages displaying active pSTAT3 signalling in IgG control treated and anti-IGF treated orthotopic murine pancreatic tumours. Data displayed as percentage of pSTAT3^+^/F480^+^ macrophages among all F480^+^ macrophages. **(C)** Quantification of the number of F480^+^ macrophages displaying active pSTAT1 signalling in IgG control treated and anti-IGF treated orthotopic murine pancreatic tumours. Data displayed as percentage of pSTAT1^+^/F480^+^ macrophages among all F480^+^ macrophages. A total of 5-8 fields of view counted/mouse tumour, n= 5 mice per treatment group, *P ≤ 0.05, **P ≤ 0.01 using Mann-Whitney U test. **(D)** Top, immunofluorescent staining of αSMA (green), pSTAT3 (red) and nuclei (blue) in formalin fixed paraffin embedded tissues from orthotopic murine PDAC tumours treated with IgG2 (control) antibody or IGF blocking antibody MEDI-573. Bottom, immunofluorescent staining of PDGFRβ (green), pSTAT1 (red) and nuclei (blue) in formalin fixed paraffin embedded tissues from orthotopic murine PDAC tumours treated IgG2 (control) antibody or IGF blocking antibody MEDI-573. Scale bar 50 µm. **(E)** Quantification of the number of αSMA^+^ fibroblasts displaying active pSTAT3 signalling in IgG control treated and anti-IGF treated orthotopic murine pancreatic tumours. Data displayed as percentage of pSTAT3^+^/αSMA^+^ fibroblasts among all αSMA^+^ fibroblasts. **(F)** Quantification of the number of PDGFRβ^+^ fibroblasts displaying active pSTAT1 signalling in IgG control treated and anti-IGF treated orthotopic murine pancreatic tumours. Data displayed as percentage of pSTAT1^+^/PDGFRβ^+^ fibroblasts among all PDGFRβ^+^ fibroblasts. A total of 5-8 fields of view counted/mouse tumour, n= 5 mice per treatment group, *P ≤ 0.05, **P ≤ 0.01 using Mann-Whitney U test. **(G)** Schematic to display experimental design of mechanistic study assessing the role of STAT signalling in controlling response to IGF blockade in BMDMS/Fibroblasts. **(H)** Immunoblotting analysis of primary murine fibroblasts and **(I)** primary murine bone-marrow derived macrophages in response to IGF blockade. Whole cell lysates were probed for both total and phosphorylated AKT, total and phosphorylated STAT3 as well as GAPDH loading control. **(J)** Quantification of *Stat1* mRNA expression levels in primary murine fibroblasts and treated with fibroblast conditioned media supplemented with either IgG control antibody (100 µg/ml), IGF-blocking antibody MEDI-573 (100 µg/ml) or recombinant IFNγ (50 ng/ml) for 1, 2, 4 or 6 hrs. n=3, **P ≤ 0.01, ****P ≤ 0.0001 using two-way ANOVA with Dunnett’s multiple comparisons test. **(K)** Quantification of *Icam1, Irf1* and *Oas2* mRNA expression levels in primary murine fibroblasts and treated with fibroblast conditioned media supplemented with either IgG control antibody (100 µg/ml), IGF-blocking antibody MEDI-573 (100 µg/ml) or recombinant IFNγ (50 ng/ml) for 6 hrs. n=3, ****P ≤ 0.0001 using two-way ANOVA with Dunnett’s multiple comparisons test. **(L)** Immunocytochemistry staining of STAT1 (red) and nuclei (blue) in primary murine fibroblasts and treated with fibroblast conditioned media supplemented with either IgG control antibody (100 µg/ml), IGF-blocking antibody MEDI-573 (100 µg/ml) or recombinant IFNγ (50 ng/ml) for 6 hrs. Bottom right, quantification of STAT1^+^ area/total cell number in IgG control, anti-IGF and IFNγ treated fibroblasts. Data displayed as fold change compared to IgG control treatment. n=3, **P ≤ 0.01,***P ≤ 0.001 using one-way ANOVA with Bonferroni’s multiple comparisons test.

In line with this hypothesis, immunofluorescent analysis of murine orthotopic PDAC tumours revealed an overall decrease in the expression of pSTAT3^Tyr705^ in both TAMs (F480^+^/pSTAT3^+^ cells) (**Figures 4A, B**) and CAFs (αSMA^+^/STAT3^+^ cells) (**Figures 4D, E**) upon IGF blockade. In turn, this marked reduction in pSTAT3 activity corresponded with an increased expression of pSTAT1^Tyr701^ in both cell types (**Figures 4A, C, D, F**).

Following this we designed further *in vitro* assays to assess whether IGF blockade inhibited the phosphorylation of STAT3 in either primary murine fibroblasts or BMDMs (**Figure 4G**). Fibroblasts or BMDMs were treated respectively with either fibroblast or BMDM conditioned media (known to contain secretory IGF ligands (3)). We found that IGF blockade inhibits phosphorylation of STAT3 in both macrophages and fibroblasts (**Figures 4H, I**). Concomitantly, IGF blockade also inhibited the phosphorylation of the upstream effector AKT (**Figures 4H, I**), in accordance with our previous work (3) and others (34).

Critically, alongside the inhibition of STAT3 phosphorylation we also observed statistically significant increases in *Stat1* gene induction in primary murine fibroblasts after 6 hours following anti-IGF treatment (**Figure 4J**), with this induction even exceeding that of fibroblasts treated with IFN-γ, a direct activator of the IFNGR/JAK/STAT1 signalling pathway (92–94). Moreover, both the indirect induction of fibroblast *Stat1* transcription through IGF blockade as well as direct induction with IFN-γ, correlated with an increased transcription of known STAT1-regulated genes *Icam1*, *Irf1* and *Oas2* (95), (**Figure 4K**), indicating an increased level of STAT1 functional activity. Finally, this increased transcriptional expression of *Stat1* upon IGF blockade correlated with an increased protein expression of STAT1 when analysing fibroblasts by immunocytochemistry (**Figure 4L**).

Taken together, these data provide mechanistic insight into how inhibition of IGF signalling in macrophages and fibroblasts leads to an inhibition of the AKT/STAT3 signalling axis, subsequent activation of STAT1 and expression of CXCL9 and CXCL10 with the dephosphorylation of STAT3 correlating with an increased expression and transcriptional activity of STAT1.

### 3.5 IGF blockade leads to increased CD8^+^ T cell recruitment towards human PDAC tumours

Having demonstrated that blocking IGF signalling increases the recruitment/trafficking of CD8^+^ T cells towards the PDAC TME via CXCL9/10 in both *in vitro* assays and in a mouse PDAC model, we aimed to validate these findings in a PDAC patient-derived *ex-vivo* model. Precision cut tumour slice (PCTS) models have been developed to allow researchers to culture fresh patient tumour tissue *ex vivo* and assess experimental drug regimens as a means of bridging the translational gap between *in vitro* and preclinical experimental findings into a more clinically relevant model of the human disease. A further advantage of the PCTS technique over other widely accepted methods of primary tissue culture, for example patient derived organoid/spheroid models is that we are able to preserve the spatial localisation of the various interacting cell types of the PDAC TME (41).

**Figure 5:**
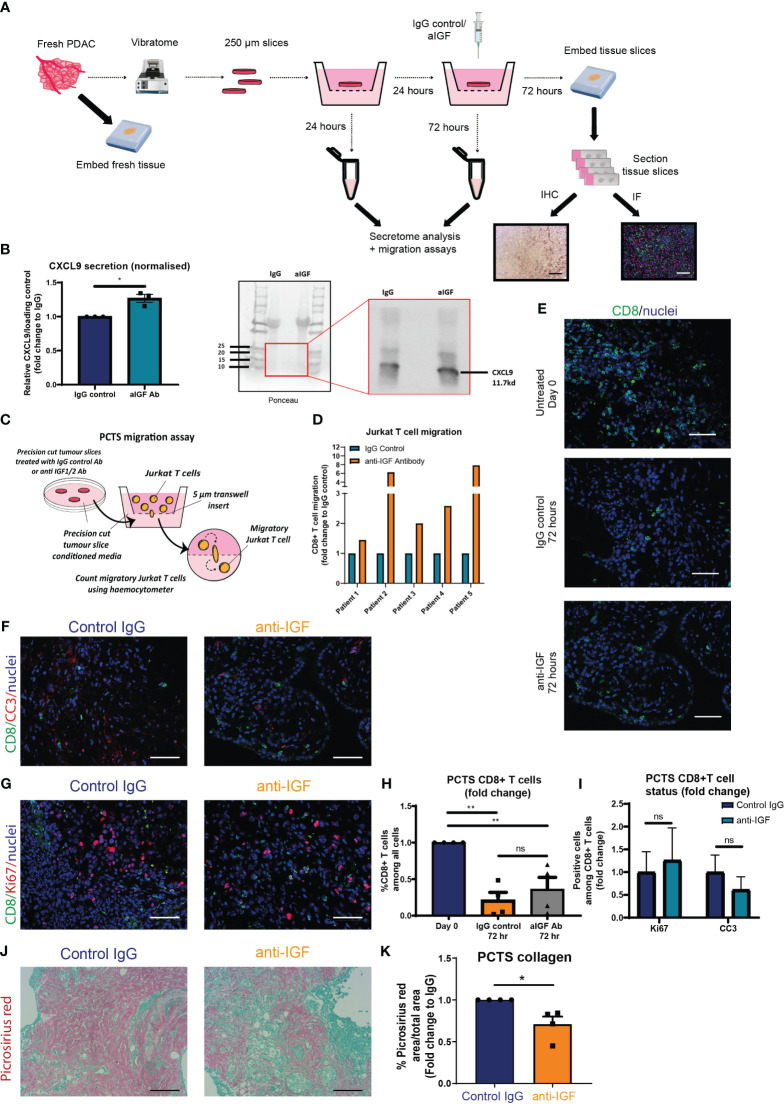
IGF blockade leads to increased CD8^+^ T cell recruitment towards human PDAC conditioned media utilising the precision cut tumour slice model **(A)** Schematic detailing the workflow for each fresh PDAC sample and generation of 250 µm precision cut tumour slices. **(B)** Left, densitometry data displaying expression of CXCL9 in conditioned media of PCTS tissue treated with IgG2 (control) antibody or IGF blocking antibody MEDI-573 for 72 hours, analysed by immunoblotting. Data displayed as fold change compared to the IgG2 control antibody of CXCL9/Ponceau loading control. n=3, *P ≤ 0.05 using one-sample t test. Right, representative immunoblotting analysis of PCTS CM, using ponceau as loading control. **(C)** Migration of Jurkat T cells through a 5 µm transwell insert towards PCTS conditioned media was measured after 15 hours using a haemocytometer. Conditioned media was generated from PCTS samples treated with IgG control antibody (100 µg/ml) or IGF-blocking antibody MEDI-573 (100 µg/ml) for 72 hours. **(D)** Data are presented separately for each patient displaying the number of migratory Jurkat T cells as a fold change compared to the IgG control treated PCTS conditioned media AFTER 15 hr. n=5. **(E)** Immunofluorescent staining of CD8 (green) and nuclei (blue) in formalin fixed paraffin embedded tissues from day 0 control PCTS samples, or PCTS samples treated with IgG2 (control) antibody or IGF blocking antibody MEDI-573 for 72 hours. Scale bar 50 µm. **(F)** Immunofluorescent staining of CD8 (green), cleaved caspase 3 (red) and nuclei (blue) in formalin fixed paraffin embedded tissues from PCTS samples treated with IgG2 (control) antibody or IGF blocking antibody MEDI-573 for 72 hours. Scale bar 50 µm. **(G)** Immunofluorescent staining of CD8 (green), ki67 (red) and nuclei (blue) in formalin fixed paraffin embedded tissues from PCTS samples treated with IgG2 (control) antibody or IGF blocking antibody MEDI-573 for 72 hours. Scale bar 50 µm. **(H)** Quantification of CD8^+^ T cells in Day 0 control, IgG control treated and anti-IGF treated PCTS samples. Data displayed as fold change of CD8^+^ T cells among all cells compared to day 0 control slices. A total of 3-4 fields of view counted/slice, n= 4 slices per treatment group, ns; P > 0.05, **P ≤ 0.01 one-way ANOVA Bonferroni’s multiple comparison *post hoc* test. **(I)** Quantification of CC3^+^ CD8^+^ T cells and Ki67^+^ CD8^+^ T cells in IgG control treated and anti-IGF treated PCTS samples. Data displayed as fold change of either CC3^+^ CD8^+^ T cells or Ki67^+^ CD8^+^ among all CD8^+^ T cells compared to IgG control treatment. A total of 3-4 fields of view counted/slice, n= 6 slices per treatment group, ns; P > 0.05 using two-way ANOVA with Bonferroni’s multiple comparisons test. **(J)** Picrosirius red staining of collagen fibres in formalin fixed paraffin embedded tissues from PCTS samples treated with IgG2 (control) antibody (top) or IGF blocking antibody MEDI-573 (bottom) for 72 hours. **(K)** Quantification of picrosirius red staining in PCTS samples. Data displayed as fold change in picrosirius red area over total area stained compared to IgG control treatment. n=4, *P ≤ 0.05 using one-sample t test.

**Figure 5A** describes the workflow for each primary PDAC sample using a protocol adapted from (41) and (42). Before proceeding with any downstream analysis, the viability of each PCTS tissue was assessed by immunohistochemistry (cleaved caspase 3 and Ki67) (**Supplementary Figure S4A**). In addition, we confirmed successful inhibition of pIGF-1R/InsR signalling within slices treated with IGF-blocking antibody as a further quality control (**Supplementary Figure S4A**). We also confirmed the presence of both αSMA expressing fibroblasts and CD206 expressing macrophages within the TME of all PCTS tissues (**Supplementary Figures S4B, C**), which are the known top producers of IGF ligands (3) and our proposed source of extracellular CXCL9/10 upon IGF blockade.

Recapitulating our *in vitro* and *in vivo* findings, we observed a modest but statistically significant increase in the expression of extracellular CXCL9 when analysing the conditioned media of PDAC patients-derived PCTS upon IGF blockade (**Figure 5B**). Moreover, we found that human Jurkat T cells migrate preferentially towards PCTS conditioned media in which the primary PDAC slices were treated with IGF-blocking antibody (**Figures 5C, D**), consolidating our *in vitro* findings utilising primary murine fibroblast conditioned media (**Figure 3Q**).

Following this, immunofluorescent analysis was used to assess the overall number and status of CD8^+^ T cells within the PCTS tissue upon IGF blockade. In accordance with our *in vivo* findings, IGF blockade does not affect the overall survival of CD8^+^ T cells within the PCTS tissue, observing similarly decreased numbers of CD8^+^ T cells within control IgG and IGF-blocking antibody-treated slices after 3 days when compared to day 0 control slices (**Figures 5E, H**). In addition, there was no difference in CD8^+^ expression of cleaved caspase 3 or Ki67 within PCTS tissue after IGF blockade (**Figures 5F, G, I**). Of note, we also observe a statistically significant decrease in the expression of collagen within PCTS tissue upon IGF blockade, as analysed by picrosirius red staining (**Figures 5J, K**). This is in accordance with our *in vivo* findings at the transcriptional level in CAFs (**Figure 3J**) and *in vitro* data at the protein level assessing collagen deposition by primary murine fibroblasts (**Supplementary Figures S5A, B**). Supporting this we also observe a tendency towards a decrease collagen deposition *in vivo*, but these data did not achieve statistical significance (**Supplementary Figures S5C, D**).

## 4 Discussion

Previously reported findings from our group and others demonstrate that stromal-derived IGFs can support tumour progression in multiple cancer types. TAM/CAF derived IGFs promote tumour cell chemoresistance to gemcitabine and paclitaxel in the case of pancreatic cancer, and TNBC respectively (3, 30). Similarly, CAF derived IGF-1 promotes cisplatin resistance in bladder cancer (33) as well as the tyrosine kinase inhibitor gefitinib in non-small cell lung cancer (NSCLC) (36). In addition, CAF-derived IGF-1 stimulates tumour cell invasion and lung metastasis in orthotopic models of breast cancer through activation of the RhoA/ROCK/p-MLC pathway (35) and TAM-derived IGFs promotes cancer cell-stemness and invasiveness in *in vitro* models of thyroid cancer (37). As such, extrinsic stromal IGF signalling plays a pleiotropic role in promoting tumour progression in a multitude of cancers and through influencing multiple cancer hallmarks.

The data presented herein describe an emerging immunomodulatory role for the IGF signalling pathway in regulating the recruitment of CD8^+^ T cells to the PDAC TME. In strong accordance with these findings, Hashimoto et al. demonstrated an increased accumulation of CD8^+^ T cells within the TME of PDAC liver metastases upon IGF blockade using intrasplenic models of PDAC liver metastasis (44). Moreover, the authors demonstrate a marked reduction in liver metastasis and prolonged survival with concomitant inhibition of the PD1/PD-L1 signalling axis, providing strong rationale for a combinatory approach that simultaneously targets the TME when designing immunotherapeutic treatment regimens.

Interestingly, these authors also observe an increase in CD8^+^ functional activity within the TME upon inhibition of IGF signalling, demonstrating an increased protein level of IFN-γ and transcriptional increases in *GZMB* encoding the serine protease Granzyme B (44). This is in direct contrast to our *in vivo* findings where we observe no changes in CD8^+^ T cell survival (cleaved caspase 3), proliferation (Ki67) or function (CD69, Granzyme B) in response to IGF blockade. In support of this *in vivo* data we also observe no differences in CD8^+^ T cell survival (cleaved caspase 3) or function (IFN-γ) *in vitro* when T cells are grown in IGF-inhibited fibroblast conditioned media. This discrepancy likely owes to the inherently vastly differing TMEs of both the PDAC liver metastatic site and the primary PDAC site (96, 97).

**Figure 6:**
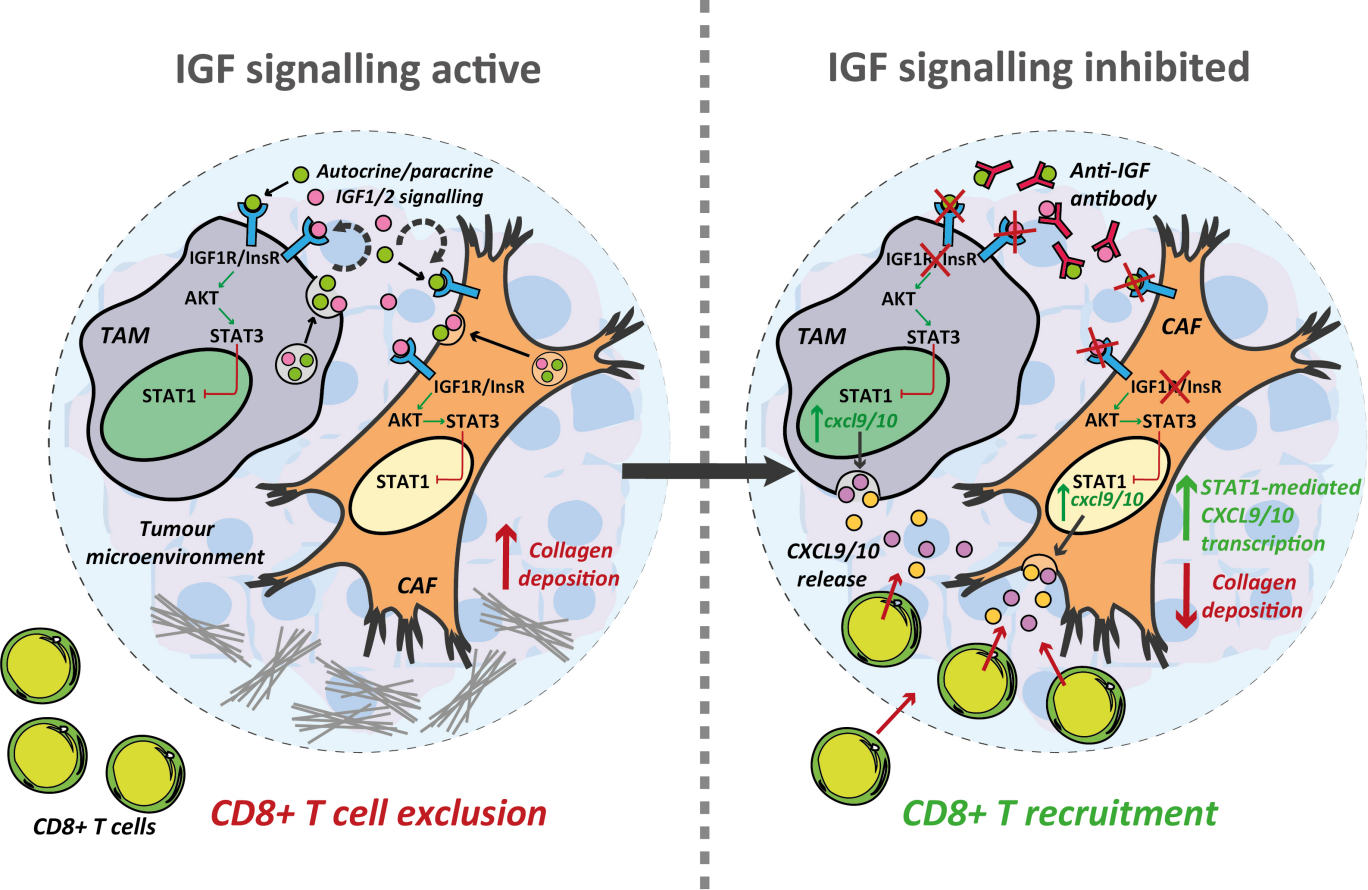
Inhibition of the IGF signalling axis facilitates T cell recruitment towards the PDAC TME. Summary schematic detailing the proposed mechanism through which IGF blockade facilitates CD8^+^ T cell recruitment towards the PDAC TME. IGF blockade inhibits STAT3 signalling in both TAMs and CAFs driving STAT1 mediated transcription of the T cell chemokines *Cxcl9/10.* Concomitantly, IGF blockade leads to a reduction in collagen deposition which may further facilitate CD8^+^ T cell infiltration into and through the PDAC TME.

Building on these findings our study explores the effects of IGF blockade on the TME at a more mechanistic level providing convincing data to show that inhibition of the IGF signalling axis promotes CD8^+^ T cell recruitment to PDAC tumours, at least in part through increased CXCL9/10 production by tumour associated macrophages and fibroblasts (**Figure 6**). IGF blockade inhibits phosphorylation/activation of STAT3, which attenuates pSTAT3 repression of STAT1, increasing its transcriptional activity and thereby inducing the expression of both *Cxcl9* and *Cxcl10*. In addition, we provide evidence to show that IGF blockade inhibits CAF collagen deposition, potentially facilitating CD8^+^ T cell infiltration into the PDAC TME (**Figure 6**).

In support of our findings, a recent study by Shang et al. has shown that inhibition of TRIB3 signalling increases CD8^+^ T cell accumulation in colorectal tumours in a similar STAT3/STAT1/CXCL10 dependent mechanism (71). The authors had previously demonstrated a role of TRIB3 in promoting STAT3 phosphorylation/activation in NSCLC (98), with targeted therapeutic degradation of TRIB3 promoting STAT1 protein stability and resultant *Cxcl10* transcription (71). This ultimately led to an increase in CD8^+^ T cell accumulation within colorectal tumours and potentiated the effects of PD-1 based immune checkpoint inhibition. With the majority of colorectal cancer patients displaying “immunologically cold” tumours and poor response to ICIs (99), this represents an exciting therapeutic angle: turning “cold” tumours “hot” through increased recruitment of CD8^+^ T cells provides a platform to increase efficacy of ICIs in the context of cancers which have traditionally shown limited response such as colorectal carcinoma and PDAC.

Indeed, the link between CXCR3 ligands, including both CXCL9 and CXCL10 in promoting anti-tumour immunity is becoming increasingly recognised, with their expression correlating with improved patient responses and sensitisation to immune checkpoint blockade in pan-cancer studies (60, 100, 101). Additionally, the role of CXCL9-expressing TAMs in the recruitment and positioning of functional CD8^+^ cytotoxic T cells has been shown to be increasingly important in orchestrating an effective anti-tumour response, and has been the focus of a recent review by Marcovecchio et al. (102). Furthermore, a recent study redefines macrophage polarity on the basis of their expression of *CXCL9* and *SPP1*, as opposed to traditional “M1” and “M2” markers, with this signature displaying prognostic significance across multiple cancer types (103).

The clinical relevance of our study is strengthened by incorporating a human *ex vivo* model of PDAC which we employ to faithfully reproduce both our *in vitro* and *in vivo* findings. However, since IGF blockade alone provides only limited efficacy, future studies should evaluate the therapeutic potential of combining IGF inhibition with ICI and chemotherapy. Given that ~50% of CD8^+^ T cells remain positive for PD-1 after IGF blockade, despite their increased intra-tumoral accumulation, a concomitant inhibition of the PD-1/PD-L1 signalling axis alongside anti-IGF treatment and chemotherapy would be a logical combinatory approach to be explored in future studies. In addition, a recent study evaluating melanoma tumours utilising multiplexed imaging mass cytometry with RNAscope *in situ* hybridisation revealed that *Cxcl9* and *Cxcl10* rich milieus also contain high densities of LAG3^+^ CD8^+^ (104). Whilst the expression of the exhaustion marker LAG3^+^ was not evaluated in the present study, this could represent another logical target of combinatory anti-IGF and ICI treatment. Given that aberrant IGF signalling is implicated in a multitude of solid malignancies, further studies should also see if this immunomodulatory role in regulating CD8^+^ T cell recruitment to tumours is conserved across multiple cancer types. Additionally, the decreases in fibroblast collagen deposition *in vitro* as well as the decreases in CAF *Col1a1* and *Col1a2* upon IGF blockade are recapitulated in our *ex vivo* culture of primary patient PDAC tissues. This could suggest an additional role for IGF signalling in regulating ECM proteins within the TME, with IGF blockade potentially facilitating CD8^+^ T cell infiltration through a decrease in collagen deposition, which should be further explored by future studies.

Overall, inhibition of the IGF signalling axis promotes TAM and CAF production of CXCL9/10 to facilitate CD8^+^ T cell recruitment to PDAC tumours. The development of strategies to enhance T cell trafficking towards the tumour is fundamental to improving therapeutic response to immunotherapy (105).

## Data availability statement

The original contributions presented in the study are included in the article/**Supplementary Material**. Further inquiries can be directed to the corresponding author.

## Ethics statement

The studies involving humans were approved by National Research Ethics (NRES) Service Committee North West – Greater Manchester REC15/NW/0477 and REC19/NW/0298. The studies were conducted in accordance with the local legislation and institutional requirements. The participants provided their written informed consent to participate in this study. The animal study was approved by UK home office. All animal experiments were performed in accordance with current UK legislation under an approved project license PPL P16F36770. Mice were housed under specific pathogen-free conditions at the Biomedical Science Unit at the University of Liverpool. The study was conducted in accordance with the local legislation and institutional requirements.

## Author contributions

PF: Data curation, Formal analysis, Investigation, Methodology, Validation, Visualization, Writing – original draft, Writing – review & editing. GB: Formal analysis, Investigation, Validation, Writing – review & editing. LI: Formal analysis, Investigation, Methodology, Writing – review & editing. MA: Investigation, Methodology, Writing – review & editing. TL: Investigation, Methodology, Writing – review & editing. MO: Resources, Writing – review & editing, Methodology. RS: Resources, Writing – review & editing. PG: Resources, Writing – review & editing. CH: Resources, Writing – review & editing. MS: Investigation, Resources, Writing – review & editing. AM: Conceptualization, Funding acquisition, Investigation, Project administration, Resources, Supervision, Visualization, Writing – original draft, Writing – review & editing.
