# Supplementary material for: Inhibition of insulin-like growth factors increases production of CXCL9/10 by macrophages and fibroblasts and facilitates CD8+ cytotoxic T cell recruitment to pancreatic tumours
Source: Front Immunol. 2024 Aug 5;15:1382538. doi: 10.3389/fimmu.2024.1382538 (PMC11334161; doi:10.3389/fimmu.2024.1382538)
Supplement: Supplementary file 1 [file DataSheet_1.docx]

Supplementary Material
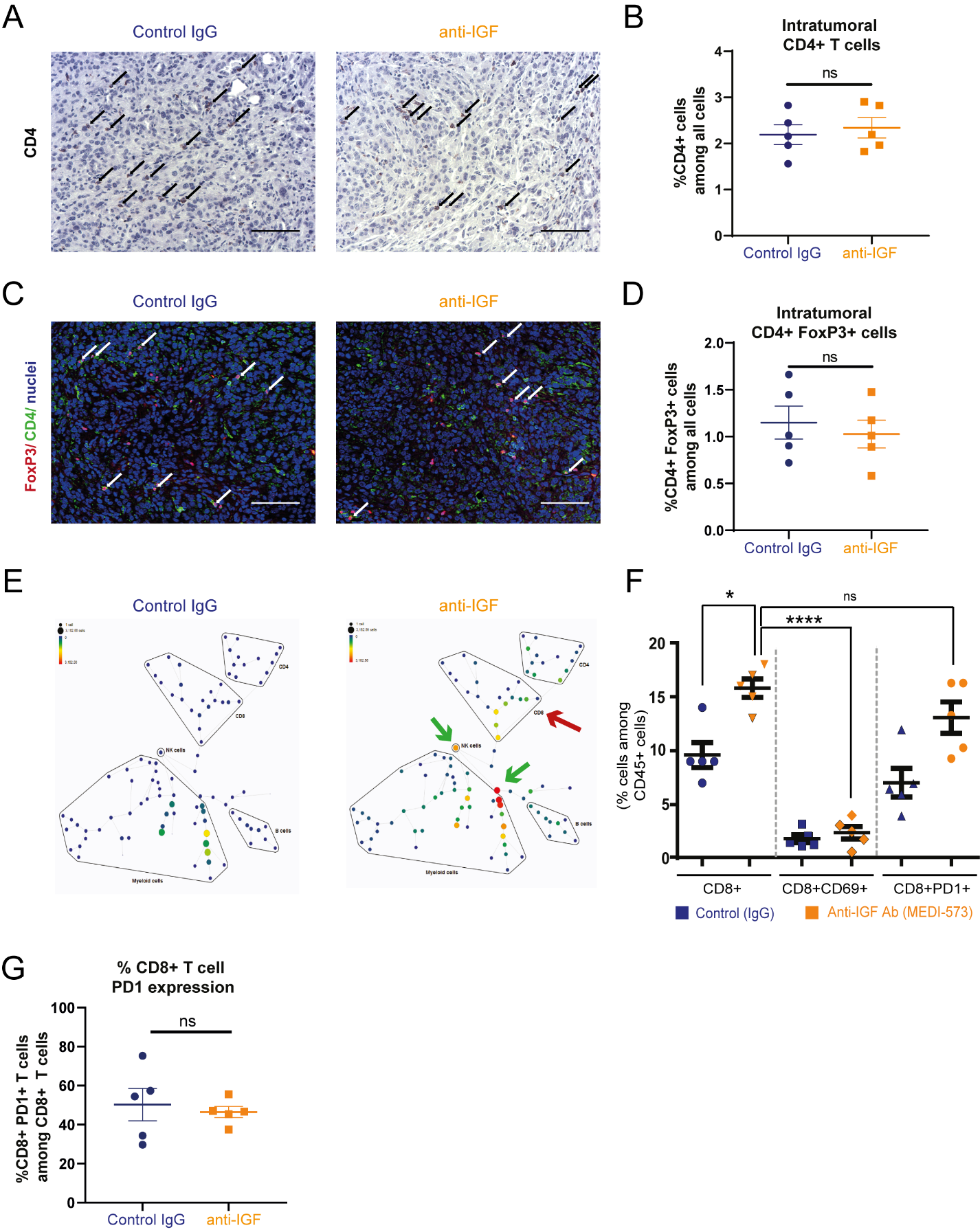


**Supplementary Figure 1.** **(A)** Immunohistochemical staining of CD4^+^ T cells in formalin fixed paraffin embedded tissues from orthotopic murine PDAC tumours treated with IgG2 control antibody or IGF-blocking antibody MEDI-573. Scale bar; 50 µm. **(B)** Quantification of CD4 staining. Data displayed as total CD4^+^ T cells among all cells. A total of 5-8 fields of view counted/mouse tumour, n = 5 mice per treatment group, ns; P > 0.05 using Mann-Whitney U test. **(C)** Immunofluorescent staining of FoxP3 (red), CD4 (green) and nuclei (blue) in formalin fixed paraffin embedded tissues from orthotopic murine PDAC tumours treated with IgG2 (control) antibody or IGF blocking antibody MEDI-573. Scale bar 50 µm. **(D)** Quantification of functionally active CD8^+^ T cells in IgG control treated and anti-IGF treated orthotopic murine pancreatic tumours. Data displayed as percentage of CD4^+^/FoxP3^+^ cells among all CD4^+^ T cells. A total of 5-8 fields of view counted/mouse tumour, n= 5 mice per treatment group, ns; P > 0.05 using Mann-Whitney U test. **(E)** SPADE (Spanning-tree Progression Analysis of Density-normalised Events) representations of CyTOF analysis performed on orthotopic murine PDAC tumours treated with IgG2 control antibody (left) or IGF-blocking antibody MEDI-573 (right). The SPADE algorithm was used to perform unsupervised clustering of cells according to their expression of 24 cell surface markers. **(F)** Quantification of CD8^+^, CD8^+^/CD69^+^ and CD8^+^/PD1^+^ T cells among CD45^+^ immune cells isolated from orthotopic murine PDAC tumours treated with IgG2 control antibody or IGF-blocking antibody MEDI-573. N=5 mice per treatment group, ns; P > 0.05, * P ≤ 0.05, **** P ≤ 0.0001 using two-way ANOVA with Bonferroni’s multiple comparisons test. **(G)** Quantification of CD8^+^ PD1^+^ T cells among all CD8^+^ T cells in IgG control treated and anti-IGF treated orthotopic murine pancreatic tumours. Data displayed as percentage of CD8^+^/PD1^+^ cells among all PD1^+^ T cells. A total of 5-8 fields of view counted/mouse tumour, n= 5 mice per treatment group, ns; P > 0.05 using Mann-Whitney U test


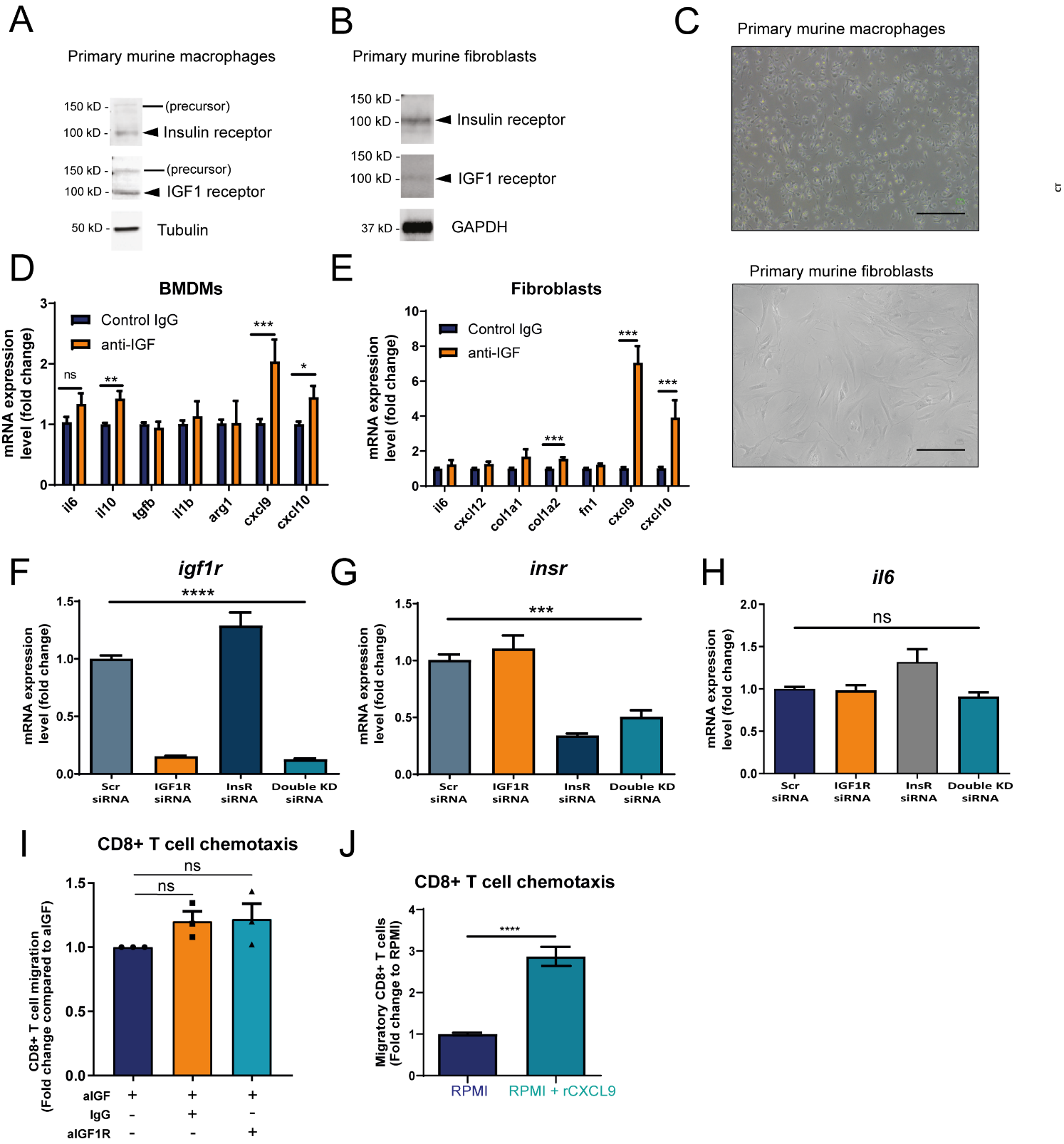


**Supplementary Figure 2. (A)** Immunoblotting analysis of primary murine bone marrow derived macrophages and **(B)** primary murine pancreatic fibroblasts. Whole cell lysates were probed for both insulin receptor and IGF1 receptor as well as either tubulin or GAPDH loading control respectively. **(C)** Top, representative brightfield image (10X magnification) of primary bone marrow derived macrophages grown in vitro for five days with mCSF-1 (10 ng/ml). Scale bar, 100 μm. Bottom, representative brightfield image (10X magnification) of primary murine pancreatic stellate cells grown in vitro for 6 days with L-glutamine (1 %). Scale bar, 100 µm. **(D)** Quantification of *Il6, Il10, Tgfb, Il1b, Arg1, Cxcl9* and *Cxcl10* mRNA expression levels in primary murine macrophages isolated from the bone marrow of wild-type C57BL/6J mice and treated with IgG control antibody (100 µg/ml) or IGF-blocking antibody MEDI-573 (100 µg/ml) for 48 hours. Expression data displayed as fold change compared to IgG control treatment. ns, *P* > 0.05; *, *P* ≤ 0.05; ***P* ≤ 0.01; ****P* ≤ 0.001 using Mann-Whitney U test **(E)** Quantification of *Il6, Cxcl12, Col1a1, Col1a2, Fn1, Cxcl9* and *Cxcl10* mRNA expression levels in primary murine fibroblasts isolated from the pancreata of wild-type C57BL/6J mice and treated with IgG control antibody (100 µg/ml) or IGF-blocking antibody MEDI-573 (100 µg/ml) for 48 hours. Expression data displayed as fold change compared to IgG control treatment. ****P* ≤ 0.001 using Mann-Whitney U test. **(F)** Quantification of *Igfr1,* **(G)** *Insr* and **(H)** *Il6* mRNA expression levels in primary murine fibroblasts isolated from the pancreata of wild-type C57BL/6J mice and treated with scrambled control siRNA (5 µM) *Igfr1* siRNA (5 µM) *Insr* (5 µM) or a combination of both *Igfr1* and *Insr* siRNAs (5 µM). Expression data displayed as fold change compared to scrambled control siRNA treatment ***, *P* ≤ 0.001; *****P* ≤ 0.0001 using one-way ANOVA with Tukey’s multiple comparisons test. **(I)** Migration of primary murine CD8^+^ T cells through a 5 µm transwell insert towards fibroblast conditioned media treated with the IGF-blocking antibody MEDI-573 (100 µg/ml). CD8^+^ T cells were treated with either goat IgG control antibody (1 µg/ml) or anti-IGF-1R antibody (1 µg/ml) and their migration measured after 15 hours using a haemocytometer. Data are presented as the number of migratory T cells as a fold change compared to the anti-IGF treated fibroblast conditioned media AFTER 15 hr, where CD8^+^ T cells had not been pretreated with either IgG or anti-IGF-1R antibody. n=3, ns *P* > 0.05 using one-way ANOVA with Tukey’s multiple comparison test. **(J)** Migration of primary murine CD8^+^ T cells through a 5 µm transwell insert towards RPMI media supplemented with/without recombinant murine CXCL9 (1000 ng/ml) was measured after 15 hours using a haemocytometer. Data are presented as the number of migratory T cells as a fold change compared to the RPMI control after 15 hr. n=3, *****P* ≤ 0.0001 using unpaired T test.


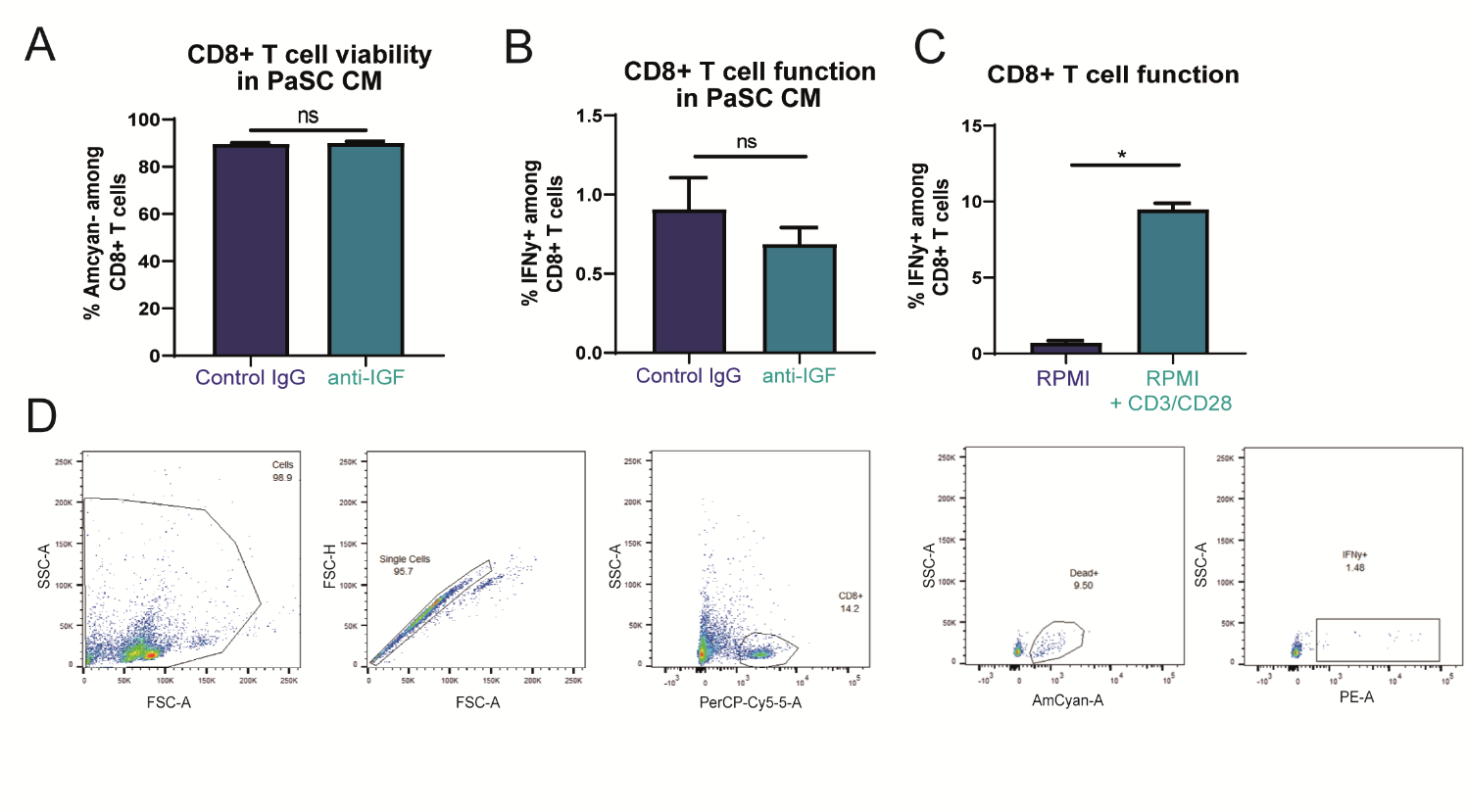


**Supplementary Figure 3.** **(A)** Flow cytometric analysis of Amcyan- CD8+ T cells cultured for 24 hours in the presence of fibroblast conditioned media. Conditioned media was generated from primary murine fibroblasts isolated from pancreata of wild-type C57BL/6J mice and treated with IgG control antibody (100 µg/ml) or IGF-blocking antibody MEDI-573 (100 µg/ml). Data are presented as the percentage of Amycan-/CD8^+^ T cells among all CD8^+^ T cells, n=4, ns; P > 0.05, using unpaired t test. (**B**) Flow cytometric analysis of IFNγ^+^ CD8^+^ T cells cultured for 24 hours in the presence of fibroblast conditioned media. Conditioned media was generated from primary murine fibroblasts isolated from pancreata of wild-type C57BL/6J mice and treated with IgG control antibody (100 µg/ml) or IGF-blocking antibody MEDI-573 (100 µg/ml). Data are presented as the percentage of IFNγ^+^/CD8^+^ T cells among all CD8^+^ T cells, n=4, ns; P > 0.05, using unpaired t test. **(C)** Flow cytometric analysis of IFNγ^+^ CD8^+^ T cells cultured for 24 hours in the presence of RPMI media supplemented with/without CD3/CD28 Dynabeads. Data are presented as the percentage of IFNγ^+^/CD8^+^ T cells among all CD8^+^ T cells. n=4, * P ≤ 0.05 using Mann- Whitney U test. **(D)** Associated FACS gating strategy used to assess the numbers of IFNγ^+^/CD8^+^ T cells


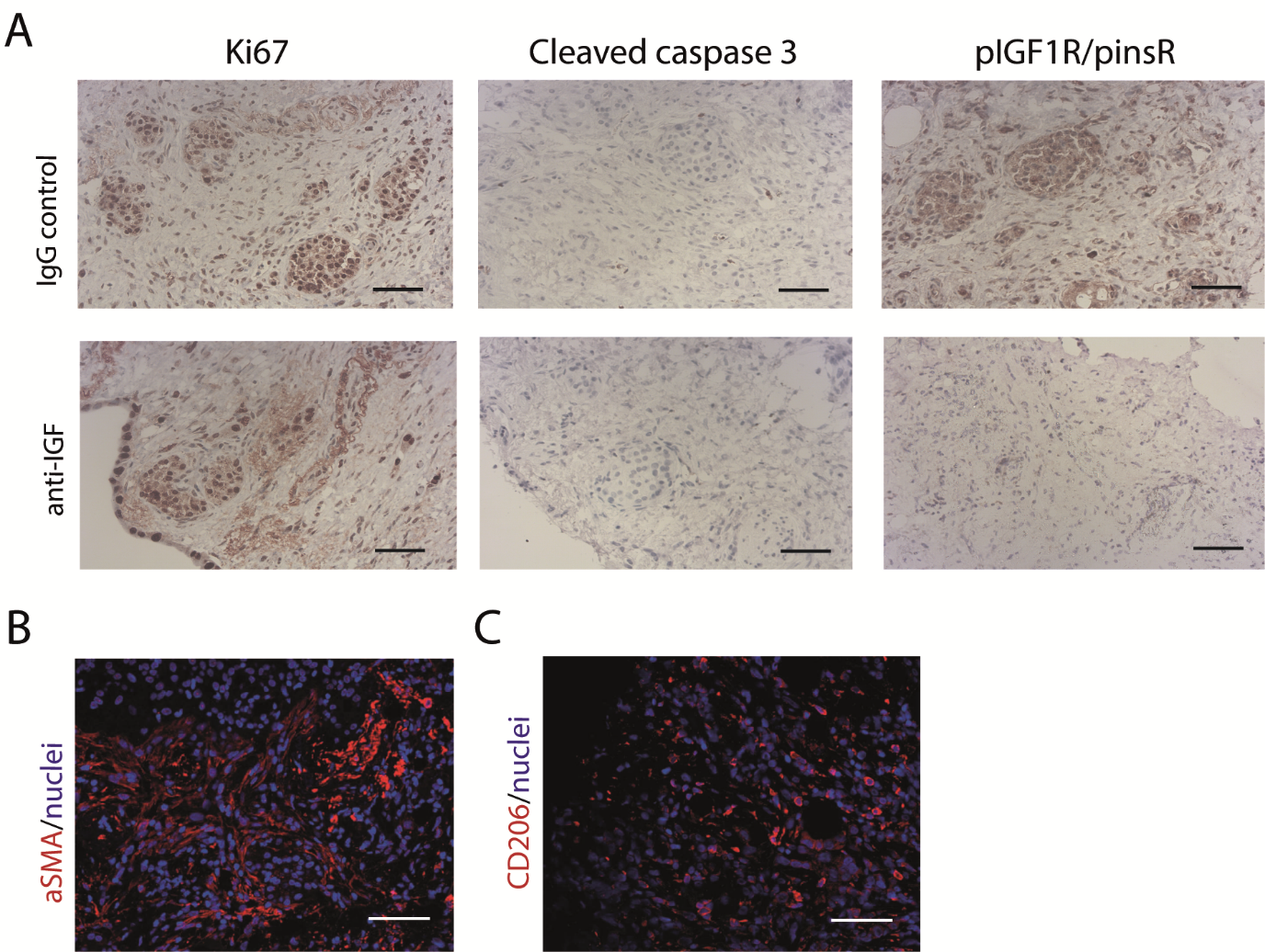


**Supplementary Figure 4. (A)** Representative immunohistochemical staining of Ki67 (left), cleaved caspase 3 (middle) and pIGFR1R/pInsR (right) in formalin fixed paraffin embedded tissues from PCTS samples treated with IgG2 (control) antibody (top) or IGF blocking antibody MEDI-573 (bottom) for 72 hours. **(B)** Representative immunofluorescent staining of αSMA (red) and nuclei (blue) in formalin fixed paraffin embedded tissues from day 0 control PCTS samples. Scale bar 50 µm. **(C)** Representative immunofluorescent staining of CD206 (red) and nuclei (blue) in formalin fixed paraffin embedded tissues from day 0 control PCTS samples. Scale bar 50 µm.


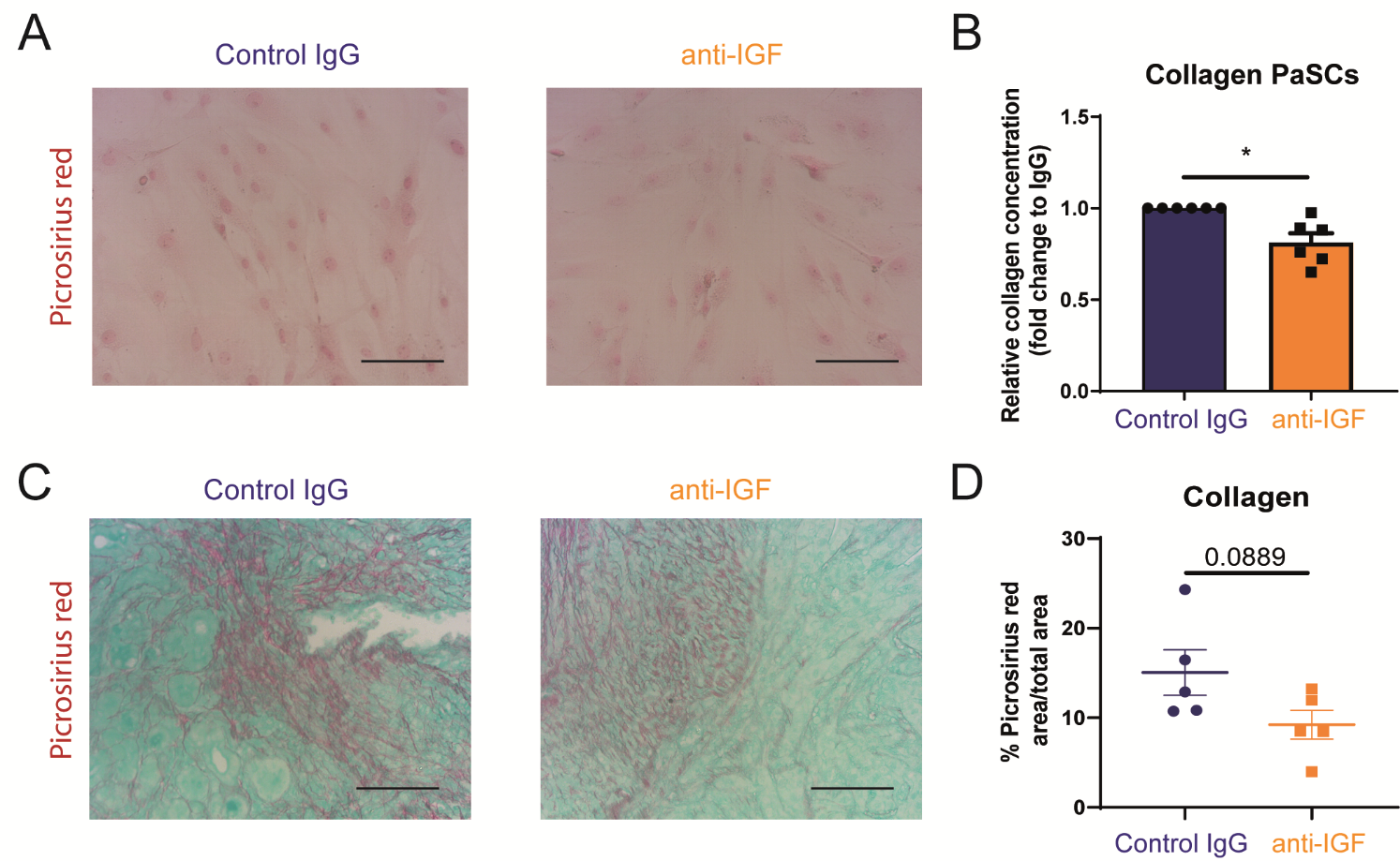


**Supplementary Figure 5. (A)** Representative brightfield images of picrosirius red staining in primary murine fibroblasts isolated from the pancreata of wild-type C57BL/6J mice and treated with IgG control antibody (100 µg/ml) or IGF-blocking antibody MEDI-573 (100 µg/ml) for 48 hours. **(B)** Quantification of picrosirius red staining. Data displayed as fold change in picrosirius red staining compared to IgG control treatment. n=6, * P ≤ 0.05 using one-sample t test. Scale bar 50 µm. **(C)** Picrosirius red staining of collagen fibers in formalin fixed paraffin embedded tissues from IgG control treated and anti-IGF treated orthotopic murine pancreatic tumours. Scale bar 50 µm. **(D)** Quantification of picrosirius red staining in IgG control treated and anti-IGF treated orthotopic murine pancreatic tumours. Data displayed as percentage picrosirius red stained area/total area in whole tumour section. n= 5 mice per treatment group. Analysed for statistical significance using unpaired t test.

**Supplementary Table 1. List of metal conjugated antibodies included in mass cytometry analysis**

| **Cell Type** | **Marker** | **Clone** | **Tag** |
| --- | --- | --- | --- |
| **Immune cells** | CD45 | 30-F11 | 147 Sm |
| **T cells** | CD3e | 145-2C11 | 152 Sm |
|  | TCRb | H57-597 | 169 Tm |
|  | CD4 | RM4-5 | 145 Nd |
|  | CD8a | 53-6.7 | 168 Er |
|  | CD25 (IL-2R) | 3C7 | 151 Eu |
|  | CD44 | IM7 | 171 Yb |
|  | CD62L (L-selectin) | MEL-14 | 160 Gd |
| **T cell activation/exhaustion** | CD69 | H1.2F3 | 143 Nd |
|  | CD279 (PD-1) | RMP1-30 | 159 Tb |
|  | CD274 (PD-L1) | 10F.9G2 | 153 Eu |
|  | CD152 (CTLA-4) | UC10-4B9 | 154 Sm |
| **Myeloid cells** | I-A/I-E (MHC class II) | M5/114.15.2 | 174 Yb |
|  | CD11b (Mac-1) | M1/70 | 148 Nd |
|  | F4/80 | BM8 | 146 Nd |
|  | CD86 | GL1 | 172 Yb |
|  | CD11c | N418 | 142 Nd |
|  | CD11b (Mac-1) | M1/70 | 148 Nd |
|  | Ly-6G | 1A8 | 141 Pr |
|  | CD115 | AFS98 | 144 Nd |
|  | CD14 | Sa14-2 | 156 Gd |
|  | Ly-6C | HK1.4 | 162 Dy |
| **B cells** | CD45R (B220) | RA3-6B2 | 176 Yb |
|  | CD19 | 6D5 | 149 Sm |
| **NK cells** | CD161 (NK1.1) | PK136 | 170 Er |
